# Supplementary material for: A novel two-factor monosynaptic TRIO tracing method for assessment of circuit integration of hESC-derived dopamine transplants
Source: Stem Cell Reports. 2021 Dec 30;17(1):159–72. doi: 10.1016/j.stemcr.2021.11.014 (PMC8758947; doi:10.1016/j.stemcr.2021.11.014)
Supplement: Document S1. Figures S1–S4 and Table S1 [file mmc1.pdf]

Stem Cell Reports, Volume *17*

Supplemental Information

A novel two-factor monosynaptic TRIO tracing method for assessment of circuit integration of hESC-derived dopamine transplants

Patrick Aldrin-Kirk, Malin Åkerblom, Tiago Cardoso, Sara Nolbrant, Andrew F. Adler, Xiaohe Liu, Andreas Heuer, Marcus Davidsson, Malin Parmar, and Tomas Björklund

Supplement | A novel two-factor monosynaptic TRIO tracing method for assessment of circuit integration of hESC-derived dopamine transplants

Author list

Patrick Aldrin-Kirk[1,3], Malin Åkerblom[1,3], Tiago Cardoso[2,3], Sara Nolbrant[2,3], Andrew F. Adler[2,3], Xiaohe Liu[1,3], Andreas Heuer[1,3], Marcus Davidsson[1,3], Malin Parmar[2,3], and Tomas Björklund[1,3*]

Affiliations

1. Molecular Neuromodulation, Department of Experimental Medical Science, Lund University, 221 84 Lund, Sweden

2. Developmental and Regenerative Neurobiology, Department of Experimental Medical Science, Lund University, 221 84 Lund, Sweden

3. Wallenberg Neuroscience Center, Lund University, Lund Sweden

Corresponding Author

* Tomas Björklund, Molecular Neuromodulation, Lund University, BMC A10 22184, Lund Sweden. E-mail: tomas.bjorklund@med.lu.se

Supplemental figures

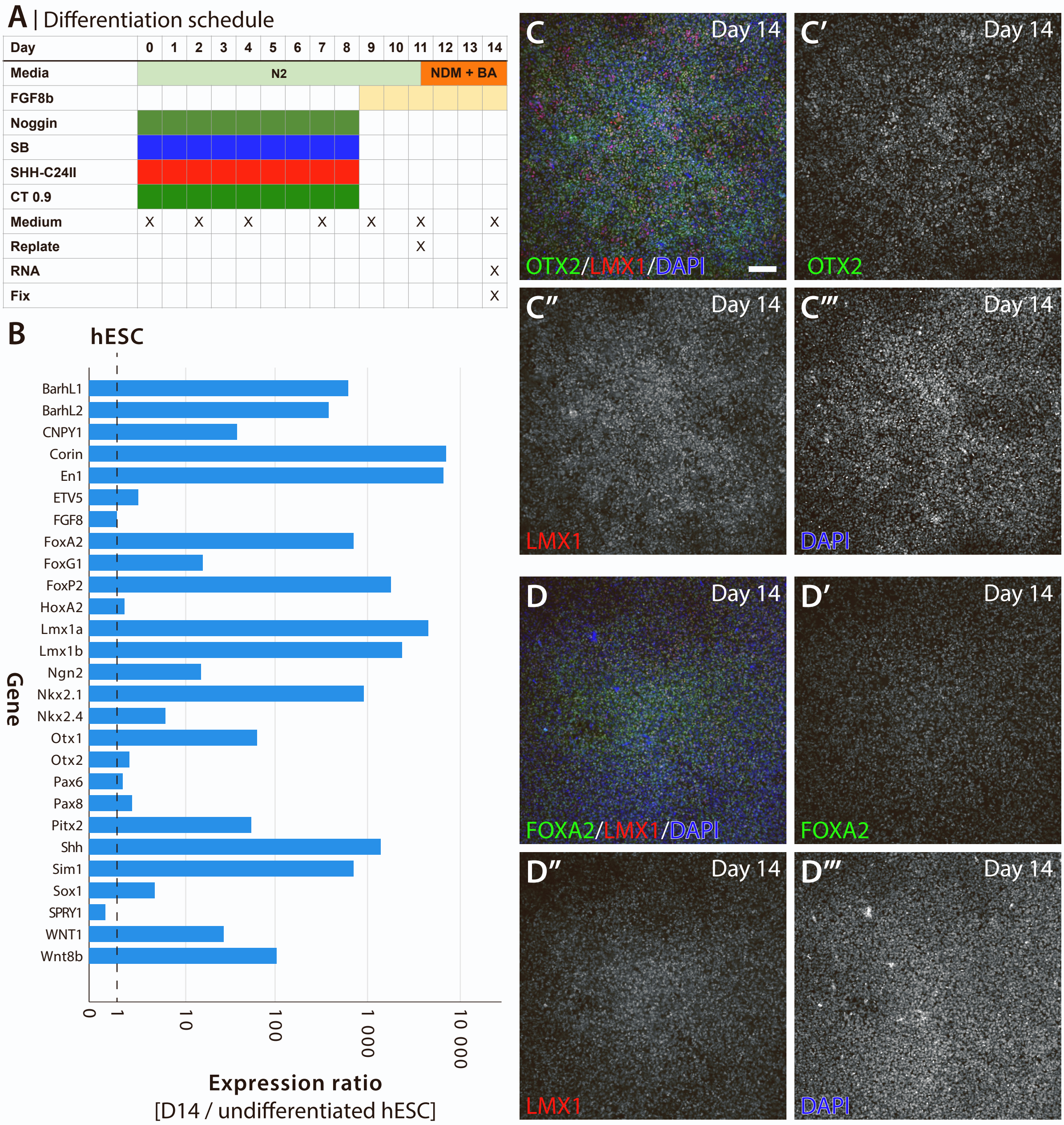

Figure S1 | Validation of the hESC differentiation protocol on LV-Cre transduced RC17 cell line, related to Figure 1

(**A**) Differentiation schedule, (**B**), quantitative RT-PCR of phenotypic markers conducted day 14 of the differentiation protocol. Transcript expression is normalized against ACTB and GAPDH and presented as fold change over undifferentiated RC17 hESCs. (**C-D**), Immunohistochemical detection of the phenotypic markers LMX1, OTX2, FOXA2 on cells fixed by Day 14 of the differentiation protocol. Scale bar in C represents 100 µm in C-D'''.

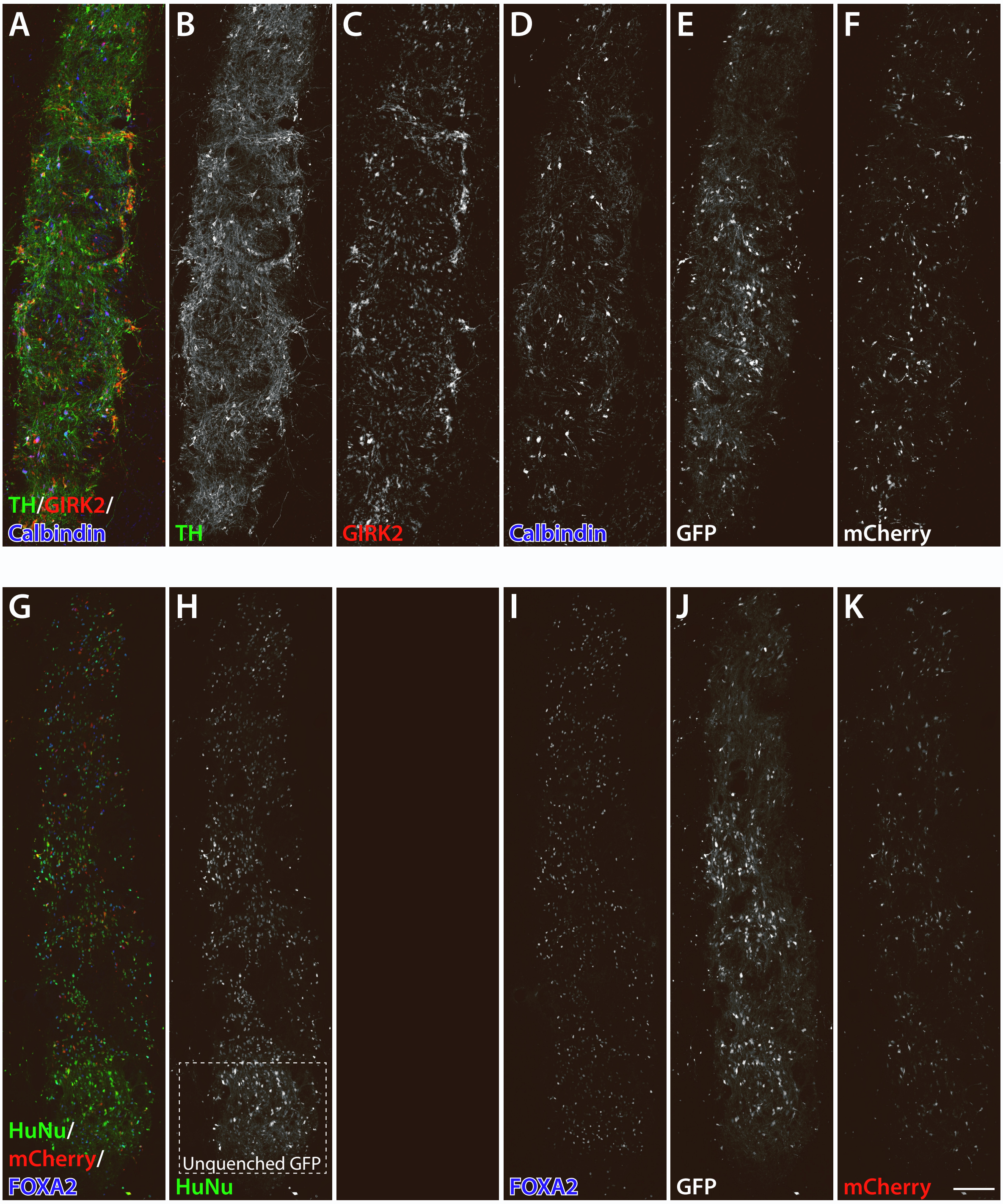

Figure S2 | Validation of phenotypic markers on matured striatal transplants, related to Figure 1

Immunohistochemical characterization phenotype and maturation of an hESC-derived graft placed in the striatum. To allow for additional fluorescent markers, biotinylated secondary Abs were used against TH (B) and HuNu (H). After the first round of imaging, the endogenous GFP (E, J) was quenched using high power 488nm LED light. After that, the sections were incubated with streptavidin-Alexa488 and re-imaged. A small region was retained unquenched for reference (dashed line in H). Scale bar in K represents 200µm.

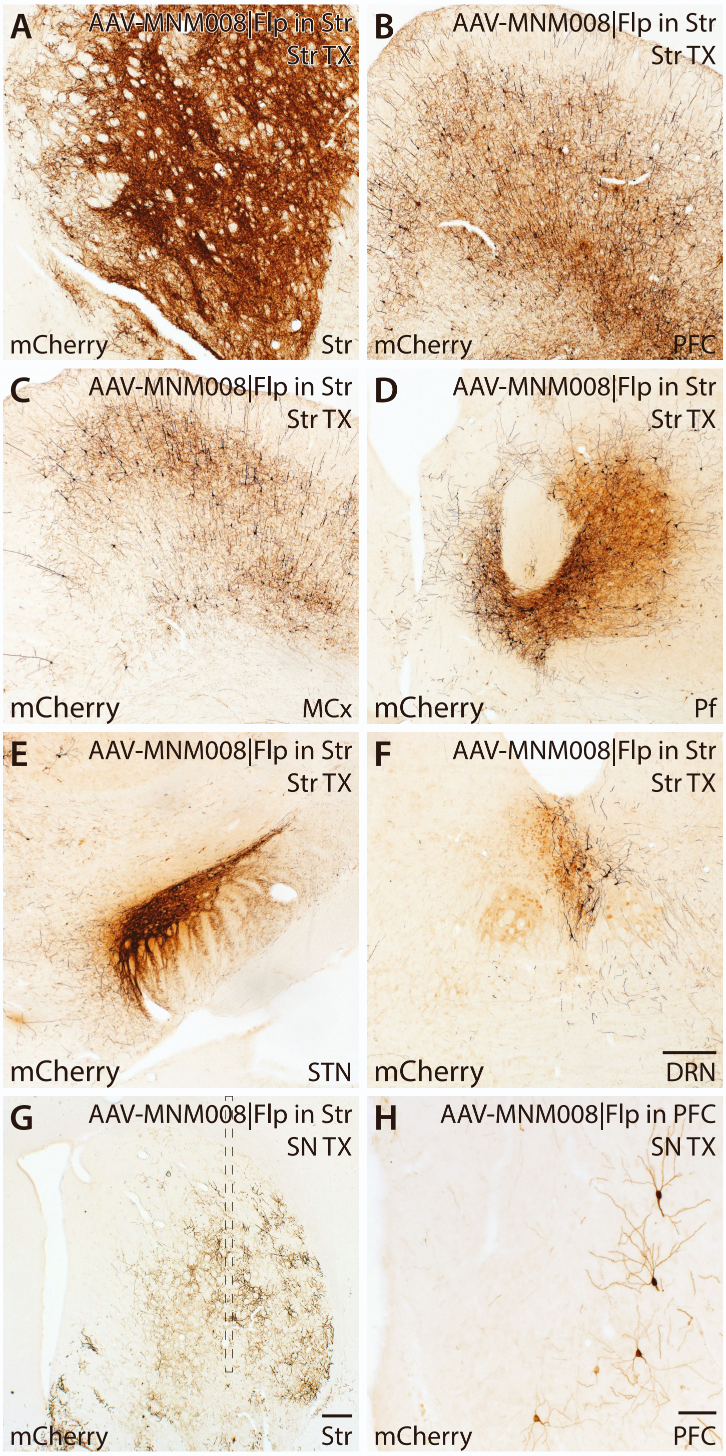

Figure S3 | Validation of AAV-based TRIO hESC-derived DA transplants in the striatum, related to Figure 1

Input to the striatal DA transplants is found in the ipsilateral striatum (Str, **A**), prefrontal cortex (PFC, **B**), the motor cortex (MCx, **C**), parafascicular nucleus (Pf, **D**), subthalamic nucleus (STN, **E**), and the dorsal Raphe nucleus (DRN, **F**). (**G**) Validation of the AAV-induced TRIO in the endogenous DA system. Injection shown as a dashed line. (**H**) Traced input nigral DA transplant with DIO-Flp injected into the PFC. Scale bar in F represents 200µm in A-F, in G represents 500µm, in H represents 50 µm.

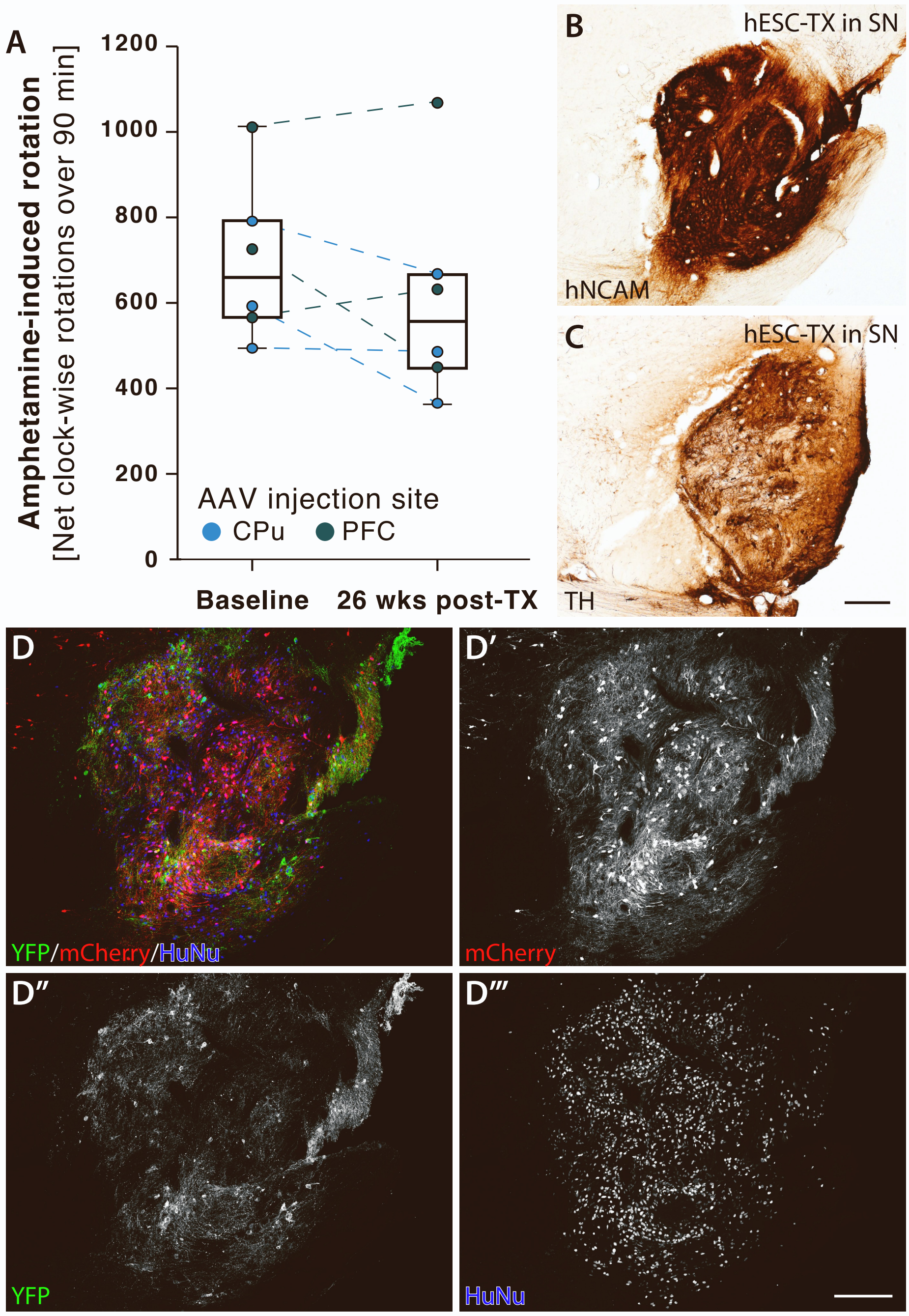

Figure S4 | Histology of nigral hESC-derived DA transplants and AAV-induced TRIO, related to Figure 3

(A) Amphetamine-induced rotation at Baseline (pre-transplantation) and 26wks post-transplantation. (**B**) Human NCAM labels all surviving hESC-derived cells in the transplant (TX). (C) A significant fraction of the surviving neurons are dopaminergic, identified using IHC against tyrosine hydroxylase (TH). (D-D'''). All YFP+ starter neurons are confirmed to be of human origin through the co-labeling with the human nuclei (HuNu) antibody. Scale bar in C represents 200µm in B-C, in D''' represents 200µm, in D-D'''.

Supplemental table 1 | qPCR primers, related to Figure 1

Gene	Full gene name	Primer sequence (fwd/rev)
ACTB	beta-actin	CCTTGCACATGCCGGAG
		GCACAGAGCCTCGCCTT
BARHL1	BarH like homeobox 1	GTACCAGAACCGCAGGACTAAA
		AGAAATAAGGCGACGGGAACAT
BARHL2	BarH like homeobox 2	GGAGATTACGAGTAGCCGTGAG
		AAGCTACGCTCCAGTTGATTGA
CNPY1	Canopy FGF signaling regulator 1	TTGGCCTCTCAAACACCATTCT
		GAGCGAAACAAAACGCAATCAC
CORIN	Corin, serine peptidase	CATATCTCCATCGCCTCAGTTG
		GGCAGGAGTCCATGACTGT
EN1	Engrailed 1	CGTGGCTTACTCCCCATTTA
		TCTCGCTGTCTCTCCCTCTC
ETV5	ETS variant 5	TCATCCTACATGAGAGGGGGTT
		GACTTTGCCTTCCAGTCTCTCA
FGF8	Fibroblast growth factor 8	ACAGCGCTGCAGAATGCCAAGT
		GAAGTGGACCTCACGCTGGTGC
FOXA2	Forkhead box A2	CCGTTCTCCATCAACAACCT
		GGGGTAGTGCATCACCTGTT
FOXG1	Forkhead box G1	TGGCCCATGTCGCCCTTCCT
		GCCGACGTGGTGCCGTTGTA
FOXP2	Forkhead box P2	ATGAGCACTCTAAGCAGCCAAT
		GTTGCAGATGCAGCAGTTCTAC
GAPDH	Glyceraldehyde-3-phosphate dehydrogenase	TTGAGGTCAATGAAGGGGTC
		GAAGGTGAAGGTCGGAGTCA
HOXA2	Homeobox A2	CGTCGCTCGCTGAGTGCCTG
		TGTCGAGTGTGAAAGCGTCGAGG
LMX1A	LIM homeobox transcription factor a	CGCATCGTTTCTTCTCCTCT
		CAGACAGACTTGGGGCTCAC
LMX1B	LIM homeobox transcription factor b	CTTAACCAGCCTCAGCGACT
		TCAGGAGGCGAAGTAGGAAC
NEUROG2	Neurogenin 2	ATCCGAGCAGCACTAACACG
		GCACAGGCCAAAGTCACAG
NKX2-1	NK2 homeobox 1	AGGGCGGGGCACAGATTGGA
		GCTGGCAGAGTGTGCCCAGA
NKX2-4	NK2 homeobox 4	AACTGCGATTCAAAACGAACCG
		GCCTCGTGGCATAATGTTACAC
OTX2	Orthodenticle homeobox 2	ACAAGTGGCCAATTCACTCC
		GAGGTGGACAAGGGATCTGA
OTX1	Orthodenticle homeobox 1	TATAAGGACCAAGCCTCATGGC
		TTCTCCTCTTTCATTCCTGGGC
PAX6	Paired box 6	TGGTATTCTCTCCCCCTCCT
		TAAGGATGTTGAACGGGCAG
PAX8	Paired box 8	ATAGCTGCCGACTAAGCATTGA
		ATCCGTGCGAAGGTGCTTT
PITX2	Paired-like homeodomain 2	AACTCTATGAACGTCAACCCCC
		CGACATGCTCATGGACGAGATA
SHH	Sonic hedgehog	CCAATTACAACCCCGACATC
		AGTTTCACTCCTGGCCACTG
SIM1	Single-minded family bHLH transcription factor 1	AAAGGGGGCCAAATCCCGGC
		TCCGCCCCACTGGCTGTCAT
SOX1	SRY-box 1	GGGAAAACGGGCAAAATAAT
		TTTTGCGTTCACATCGGTTA
SPRY1	Sprouty RTK signaling antagonist 1	GCCCTGGATAAGGAACAGCTAC
		GCCGAAATGCCTAATGCAAAGA
WNT1	Wnt family member 1	GAGCCACGAGTTTGGATGTT
		TGCAGGGAGAAAGGAGAGAA
WNT8B	Wnt family member 8B	CTAGTGGGGCAATGACTTTCCT
		TTCCTAGACCTTCGGGGTATGT
