# Supplementary material for: A novel two-factor monosynaptic TRIO tracing method for assessment of circuit integration of hESC-derived dopamine transplants
Source: Stem Cell Reports. 2021 Dec 30;17(1):159–72. doi: 10.1016/j.stemcr.2021.11.014 (PMC8758947; doi:10.1016/j.stemcr.2021.11.014)
Supplement: Document S2. Article plus Supplemental information [file mmc2.pdf]

Stem Cell Reports
Resource
OPEN ACCESS

# A novel two-factor monosynaptic TRIO tracing method for assessment of circuit integration of hESC-derived dopamine transplants

Patrick Aldrin-Kirk,[1,3] Malin Åkerblom,[1,3] Tiago Cardoso,[2,3] Sara Nolbrant,[2,3] Andrew F. Adler,[2,3] Xiaohe Liu,[1,3] Andreas Heuer,[1,3] Marcus Davidsson,[1,3] Malin Parmar,[2,3] and Tomas Björklund[1,3,*]
[1]Molecular Neuromodulation, Department of Experimental Medical Science, Lund University, BMC A10, 221 84 Lund, Sweden
[2]Developmental and Regenerative Neurobiology, Department of Experimental Medical Science, Lund University, 221 84 Lund, Sweden
[3]Wallenberg Neuroscience Center, Lund University, Lund, Sweden
*Correspondence: tomas.bjorklund@med.lu.se
https://doi.org/10.1016/j.stemcr.2021.11.014

## SUMMARY

Transplantation in Parkinson's disease using human embryonic stem cell (hESC)-derived dopaminergic (DA) neurons is a promising future treatment option. However, many of the mechanisms that govern their differentiation, maturation, and integration into the host circuitry remain elusive. Here, we engrafted hESCs differentiated toward a ventral midbrain DA phenotype into the midbrain of a preclinical rodent model of Parkinson's disease. We then injected a novel DA-neurotropic retrograde MNM008 adeno-associated virus vector capsid, into specific DA target regions to generate starter cells based on their axonal projections. Using monosynaptic rabies-based tracing, we demonstrated for the first time that grafted hESC-derived DA neurons receive distinctly different afferent inputs depending on their projections. The similarities to the host DA system suggest a previously unknown directed circuit integration. By evaluating the differential host-to-graft connectivity based on projection patterns, this novel approach offers a tool to answer outstanding questions regarding the integration of grafted hESC-derived DA neurons.

## INTRODUCTION

Cell transplantation in Parkinson's disease using human fetal ventral mesencephalon (VM) tissue has been pursued in the clinic over the last four decades (Barker et al., 2015). New cell sources such as human embryonic stem cells (hESCs) (Kim et al., 2020; Parmar et al., 2020) or induced pluripotent stem cells (Takahashi, 2020) are currently being explored, and multiple international consortia are actively working toward new clinical trials (Doi et al., 2020; Kim et al., 2021; Kirkeby et al., 2017; Xiong et al., 2021). Systematic optimization of the differentiation protocol has significantly improved the survival, maturation, and purity of dopaminergic (DA) neurons (Doi et al., 2020; Kim et al., 2021; Kirkeby et al., 2012, 2017; Kriks et al., 2011; Xiong et al., 2021). Recent studies have also elucidated how vital the specific DA subtype is for the transplant's therapeutic potential (Grealish et al., 2010; Kirkeby et al., 2017). Advances in recent years regarding the modulation of transplants (Aldrin-Kirk et al., 2016; Chen et al., 2016; Steinbeck et al., 2015) have again invigorated the field and enabled experimental studies of factors that govern a successful restoration of the basal ganglia circuitry (Bjorklund and Parmar, 2020).

The entire mammalian DA system consists of 16 distinct populations, of which 9 (A8–A16) are located in the midbrain and forebrain. Two DA neuron populations are responsible for most DA input to the basal ganglia; the substantia nigra (SN, A9) and the ventral tegmental area (VTA, A10). The A9 neurons regulate mainly the motor circuits and the A10 the limbic circuits (Bjorklund and Dunnett, 2007). Earlier studies have shown that DA transplants provide significantly higher therapeutic potential if derived from A9 neurons than those derived from A10 neurons (Grealish et al., 2010). Current hESC differentiation protocols aim to produce high-purity midbrain DA neurons (Grealish et al., 2014; Niclis et al., 2017; Nolbrant et al., 2017). While they are enriched in GIRK2 expressing DA neurons, the grafts contain DA neurons that innervate both A9 and A10 target regions (Grealish et al., 2014; Xiong et al., 2021). A second much less studied parameter is circuit reconstruction. To date, most transplantation studies have used ectopic transplant placement into the striatum (Str). However, homotopic graft placement (into the SN) holds the potential to provide complete circuit repair and form all the appropriate afferent connections of the intact nigrostriatal system.

The tracing the relationship between input and output (TRIO) approach is a recent refinement of the monosynaptic rabies tracing method. (For details of rabies tracing, see the methods section and Grealish et al. [2015] and Wickersham et al. [2007]). TRIO depends on so-called starter cells, defined by their axonal projections to a brain region of interest (Schwarz et al., 2015). Canine adenovirus (CAV-2) was used for this initial retrograde labeling in the original implementation, which has practical, immunologic limitations and has, to our knowledge, not been shown to function in human DA cells. In this study, we have modified the TRIO approach to be entirely adeno-associated virus (AAV) induced. We have recently engineered several novel AAV capsids with a high affinity for synaptic transduction and retrograde axonal transport in


This is an open access article under the CC BY license (http://creativecommons.org/licenses/by/4.0/).

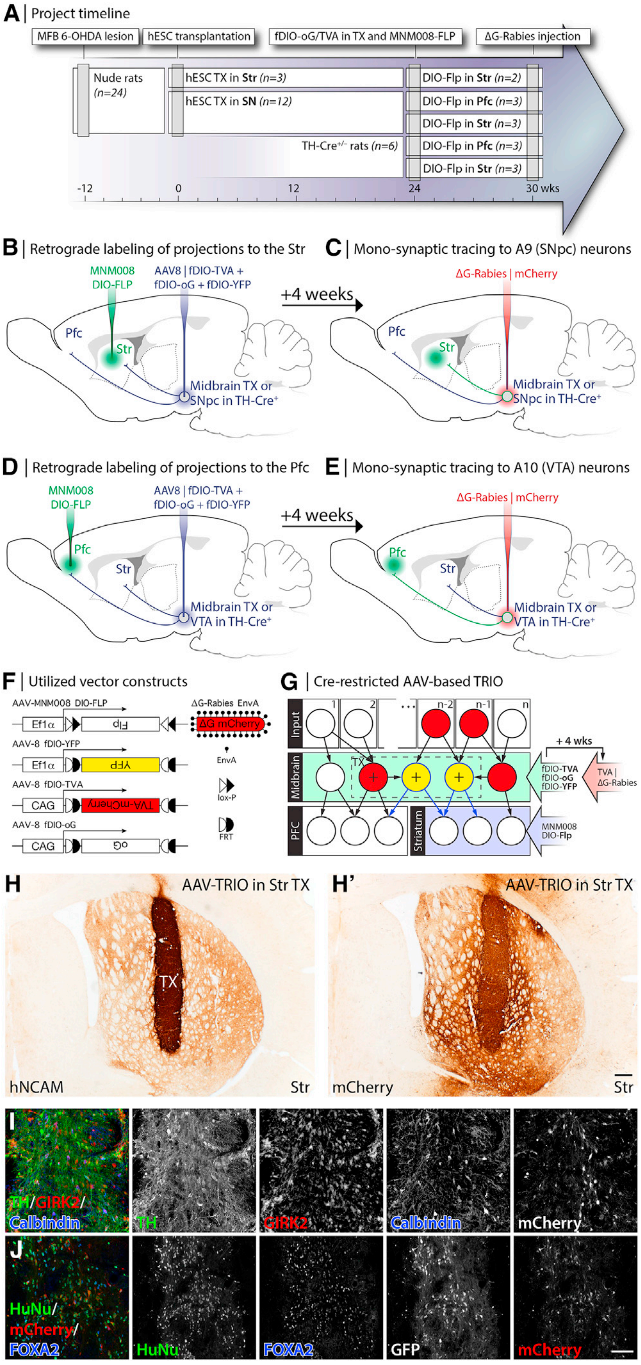


**Figure 1. Experimental design for retrograde induction of rabies tracing and the principle behind the AAV-based TRIO approach**

(A) Project timeline. For details, see the Experimental design section.

(B–E) Injection paradigms to restrict retrograde tracing to either endogenous A9 (SNpc) or A9-type graft neurons (B and C) or to endogenous A10 (VTA) or A10-type graft neurons (D and E).

(F) Vector constructs used in the study.

(G) Schematics of the selective retrograde tracing in grafted animals achieved through the Cre-restricted AAV-based TRIO approach. The schema shows the group in (B and C) targeting the A9 (SNpc-type) grafted neurons with axonal projections to the Str. The retrogradely transported MNM008 | DIO-Flp vector is injected into the Str. The Flp will only be activated in the Cre$^{+}$ neurons (large + in cells). fDIO genes will then turn on and allow for the synthesis of trans-synaptically-competent rabies virus, which is transported to all afferent neurons within the midbrain and in input regions. The end state is that the starter (A9-type) neurons are YFP$^{+}$ (pseudo-colored green) and mCherry$^{+}$ (Red), while connected afferent neurons will only be mCherry$^{+}$.

(H) An hESC graft ectopically placed in the Str, visualized using hNCAM DAB (brown) immunohistochemistry (IHC), specific to the human cell origin.

(H′) mCherry DAB IHC expressed from the SADΔG-mCherry rabies virus injected into the TVA$^{+}$ and oG$^{+}$ graft.

(I and J) Immunohistochemical characterization of the phenotype and maturation of an hESC-derived Syn1-Cre graft placed in the Str. Scale bar in (H′) represents 200 μm in (H and H′), and in J represents 100 μm in (I and J).

diverse neuronal subpopulations. One of these capsids contains an insertion of a small CAV-2–derived peptide (MNM008). This capsid has high efficiency for retrograde transport from the Str to the DA neurons in the SN pars compacta (SNpc) in rats and to hESC-derived DA neurons grafted to the Str (Davidsson et al., 2019).

In the present study, we use the MNM008 capsid for Cre-restricted, AAV-induced TRIO to uniquely assess the circuit integration of the two major hESC-derived A9 and A10 DA neuron subtypes in the grafts. The cells are identified based on their specific projection patterns to the Str and prefrontal cortex (PFC), respectively. Using this technique to infect the nigral DA transplants from their axon terminals, we show that even the adult brain, subjected to a Parkinson's disease model, is able to regenerate functional and precise connections. The patterns of host afferents are distinctly different between A9- and A10-type neurons in the transplant, and the connections closely resemble those in the intact brain.

## Experimental design

We designed this study to evaluate the differential host-to-graft connectivity between subtypes of grafted tyrosine hydroxylase (TH)-expressing DA neurons based on their projection patterns to either the PFC, the PFC (A10 phenotype), or to the Str (A9 phenotype) (timeline in Figure 1A). Twenty-four athymic nude rats were unilaterally lesioned with 6-OHDA. Rats with a complete lesion (n = 15) were grafted with Synapsin-Cre–expressing hESCs differentiated *in vitro* to a caudal VM fate known to generate DA-rich transplants (Nolbrant et al., 2017) into either the Str (n = 3) or the SN (n = 12). The grafts were then left to mature for 26 weeks to allow for target-specific axonal outgrowth to the forebrain and the formation of mature synapses (Cardoso et al., 2018). The rats then received stereotactic

injections into the Str (n = 6) (Figures 1B and 1C) or the PFC (n = 6) (Figures 1D and 1E) with the retrogradely transported AAV-MNM008 vector. This vector contained a construct for Cre-recombinase inducible flippase (double-floxed, inverted orientation, or DIO-Flp) (Figure 1F). In the same surgical session, the rats were injected with a mix of AAVs into the grafts, containing constructs for Flp-induced TVA receptor (fDIO-TVA), optimized rabies glycoprotein (fDIO-oG), and YFP (Figure 1F). Using this setup, only those grafted neurons that projected to either the PFC or the Str would express the three components: TVA receptor and rabies glycoprotein, respectively necessary for infection by recombinant rabies virus and subsequent transsynaptic spread (Figure 1G), as well as YFP to visualize these starter cells. The rats with striatal grafts (n = 3) were injected with all AAV constructs directly into the graft and served as a positive control for the serial induction of the tracing genes.

In parallel, intact TH-Cre knock-in rats (Brown et al., 2013) were used to assess connectivity in the host DA system for direct comparison with the results obtained from hESC-derived midbrain grafts. The TH-Cre rats were injected at the same coordinates with the retrogradely transported AAV-MNM008 vector containing the Cre-induced Flp construct either into the PFC (n = 3) or the Str (n = 3). In the same surgical session, AAVs were injected into the SN containing Flp-inducible TVA receptor, rabies glycoprotein, and YFP, rendering the host TH neurons that project to PFC and Str to serve, respectively, as starter cells for monosynaptic rabies tracing.

At four weeks after AAV injection, all animals were stereotactically injected with the SADΔG-mCherry strain of recombinant rabies virus into the site of DA neurons (graft or host SN/VTA) (Figures 1F and 1G). This avian envelope protein (EnvA)-pseudotyped virus is only able to initially infect cells expressing the TVA receptor. The optimized glycoprotein, oG, is supplied in trans from the AAV for trans-synaptic transfer. Therefore, this approach will only label presynaptic neurons (with mCherry) connected to cells expressing the TVA and the oG (which are also YFP labeled). Thus, mCherry-positive, YFP-negative neurons outside the graft/SN represent traced cells having formed monosynaptic contacts with starter cells ($Cre^+$ neurons in the graft or TH-Cre host SN, respectively), which themselves project to either the PFC or Str (Figure 1G). Animals were sacrificed seven days after the injection of the rabies virus to avoid rabies-induced cytotoxicity. Postmortem, scanned histological sections were aligned with the Paxinos and Watson rat brain atlas (Paxinos and Watson, 2005) and inserted into a three-dimensional (3D) representation of the brain. Individual traced cells ($mCherry^+$) were marked into the correct location and summed up based on anatomical location.

## RESULTS

### *In vitro* differentiation of hESCs produce DA neurons of a ventral midbrain phenotype

The differentiation protocol we used (Nolbrant et al., 2017) produces accurate VM progenitor patterning without any apparent influence of the lentiviral (LV)-derived Syn1-Cre expression (Figure S1). When transplanted into the Str and nigra they developed into mature neuronal transplants. A majority of the transplanted cells expressed the nigral DA marker GIRK2 (Figures 1I and S2) and a subset of these also expressed Calbindin, a marker of either A10-type neurons or a lateral population of A9-type neurons (Figure 1I) (Reyes et al., 2012). Their VM identity was confirmed by expression of FOXA2 (Figure 1J) and the transplants were rich in TH-expressing neurons (Figure 1I). This finding confirms that the transplants are of a ventral midbrain origin and that they likely contain both A9-type and A10-type DA neurons. However, to unequivocally separate them into the subtypes, these cell groups must be identified based on their target innervation patterns.

### Application of the TRIO-based tracing method to the endogenous midbrain DA system

First, we aimed to validate the technique and at the same time generate a reference of the monosynaptic input to the two major populations of the endogenous midbrain DA system: The nigrostriatal A9 population located in the SN and the mesolimbic A10 population, located in the VTA. The starter cells in the SN or VTA ($Cre^+$ and $Flp^+$, representing A9 or A10 neurons) expressed both $YFP^+$ and $mCherry^+$ in the SN or VTA (Figures 2B and 2B′). The $YFP^+$ axons terminate around the injection tract in the forebrain (Figures 2A and 2A′), with the $YFP^+$ neurons also being $TH^+$ as expected (Figures 2A, 2A‴, 2B, and 2B‴). The traced cells, expressing $mCherry^+$ alone, represent neurons with monosynaptic afferents inputs to the starter cells and were here seen both in the Str and in the midbrain (Figures 2A, 2A″, 2B, and 2B″). Sparse neuronal labeling was found in many distant afferent neurons, including the PFC, the anterior cingulate cortex (ACC, Figure 2C), the subthalamic nucleus (STN) (Figure 2D), the hypothalamus (Figure 2E), and the dorsal Raphe nucleus (DRN, Figure 2F). (For quantification of all afferents, see Figure 4).

### AAV and monosynaptic rabies-based tracing from intrastriatal hESC transplants

Next, we applied the technique to intrastriatal hESC-derived DA transplants that expressed Cre-recombinase under the pan-neuronal Synapsin-1 promoter. In this group, the AAV vectors required for monosynaptic rabies tracing (Figure 1F) were injected directly into the

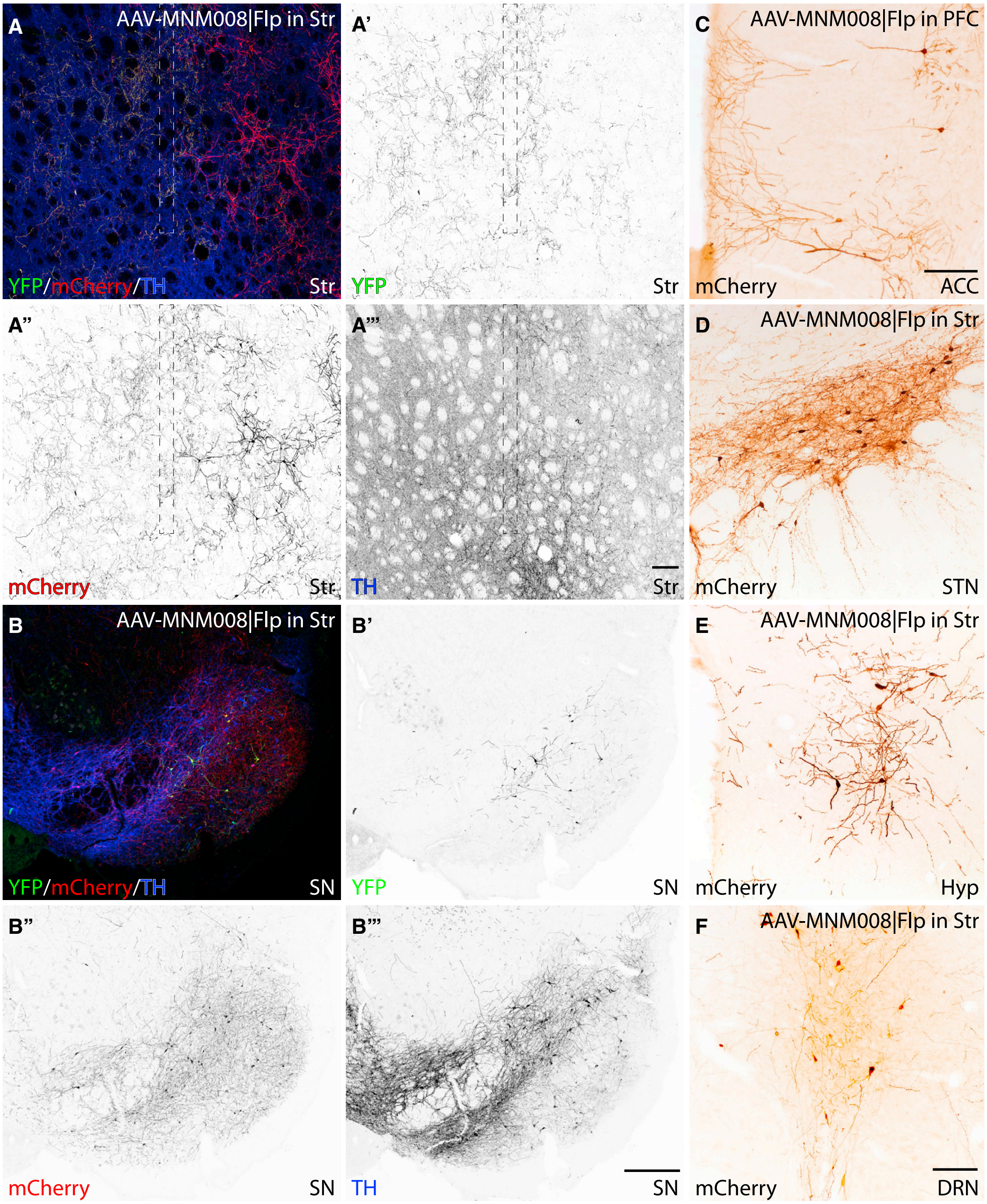


**Figure 2. AAV and monosynaptic rabies-based tracing from host DA neurons**

(A–B‴) Validation of the Cre-restricted AAV-based TRIO approach in intact TH-Cre rats. The MNM008 | DIO-Flp vector was injected into the dorsolateral Str (needle tract shown as a dashed line in (A). Starter cells and their projections are $YFP^+$ (A′ and B′) and are all $TH^+$ (A‴ and B‴). Both starter cells and traced cells are $mCherry^+$ (A′ and B″).

(C–F) *In vivo* generated rabies virus in the SN is transported to afferent neurons. Afferent neurons are found in other regions as well, including the anterior cingulate cortex (ACC [C], the STN [D], hypothalamus [Hyp] [E] and the DRN [F]). Scale bar in (A‴) represents 200 μm in (A–A‴), in (B‴) represents 200 μm in (B–B‴), in (C) represents 200 μm, in (F) represents 200 μm in (D–F).

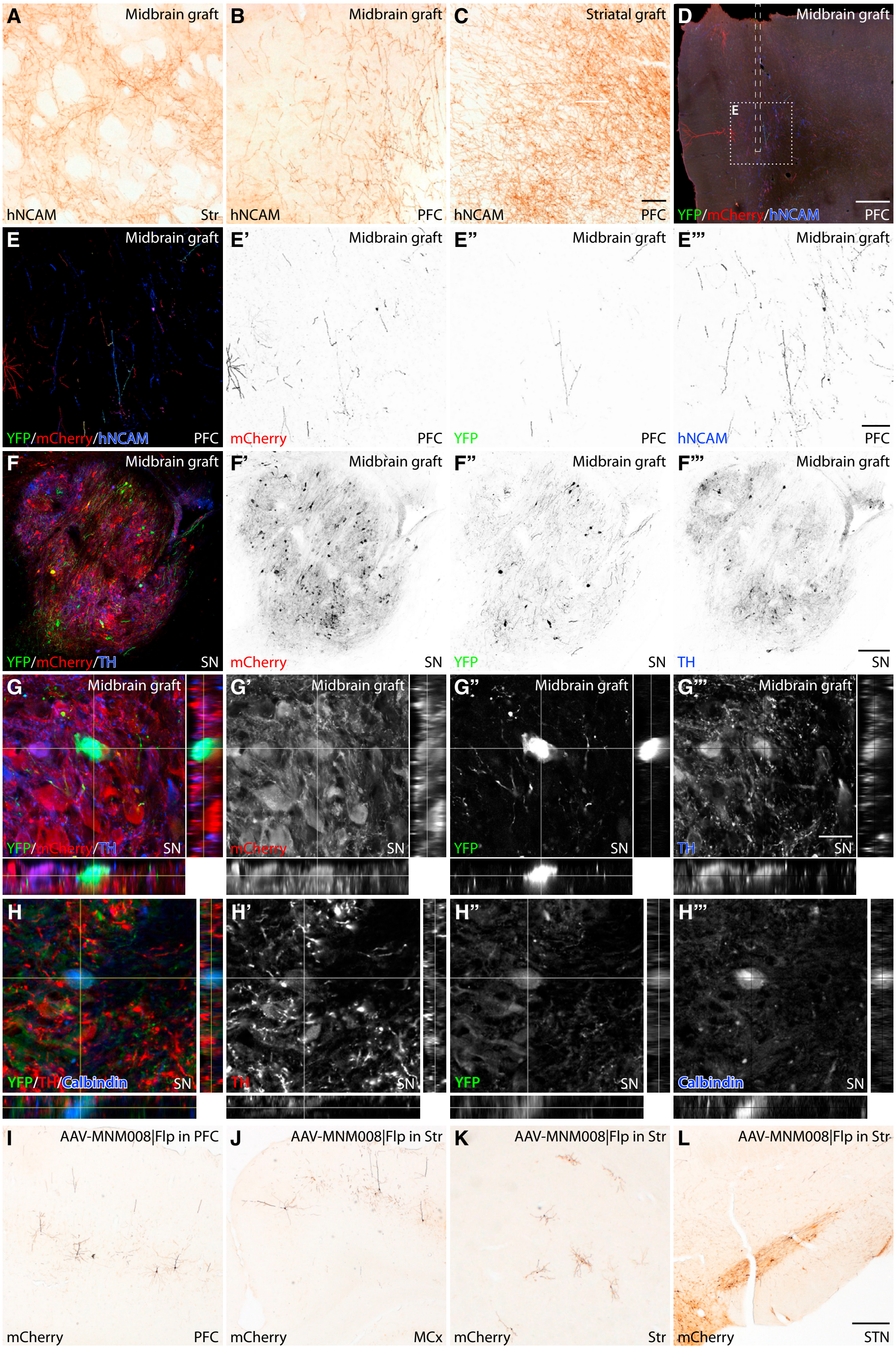


(legend on next page)

transplant. Four weeks after AAV injection, the animals received the SADΔG-mCherry rabies virus into the transplant. At six months after transplantation, the hESC transplant had developed dense innervation of most of the Str, as seen from the human neural cell adhesion molecule (hNCAM) staining, specific to the human cell origin (Figure 1H). In the traced host cells, dense mCherry immunoreactivity was observed in the transplant and the surrounding ipsilateral Str (Figure 1H′). Intense labeling was seen in all expected input regions (Figure S3), including the prefrontal and motor cortices (Figures S3B and S3C), parafascicular and subthalamic nuclei (Figures S3D and S3E), and the DRN (Figure S3F).

### Long-term intranigral graft survival and re-innervation of A9 and A10 DA target areas in the Str and PFC

To investigate the host input to A9- and A10-grafted hESC-derived DA neurons grafted to the SN in 6-OHDA lesioned nude rats, we first histologically assessed the long-term survival of grafts based on the distribution of their axonal projections. The same Synapsin-Cre–expressing hESC-derived DA progenitors, as used above, were transplanted to the DA-depleted SN. Graft survival was assessed after twenty-six weeks, a time point where these grafts have previously shown sufficient maturation to provide functional recovery and to have completed axonal outgrowth along the medial forebrain bundle and nigrostriatal pathway (Adler et al., 2019; Cardoso et al., 2018; Grealish et al., 2014). We visualized reinnervation through DAB and immunofluorescence stainings of the human-specific hNCAM marker within the midbrain of grafted animals. Histological analysis revealed surviving grafts in most animals, though they were variable in size (Figure S4). Animals with surviving grafts transplanted to the SN displayed hNCAM positive projections in both target regions within the Str and PFC (Figures 3A and 3B). As expected from earlier studies, the innervation density in the PFC was lower from intranigral grafts than that from transplants placed in the Str (Figure 3C).

### Visualization of monosynaptic inputs to intranigral grafts neuron compartments based on their striatal or PFC projections

To investigate whether transplanted neurons of the A9 subtype (SN type) differed in their afferent inputs from the A10 (VTA type) neurons, we repeated the procedure conducted on the endogenous DA system. We used AAV8 | fDIO-TVA, and AAV8 | fDIO-oG injected into the graft, together with the AAV-MNM008 | DIO-Flp injected into the Str (labeling A9-like graft neurons) or PFC (labeling A10-like graft neurons) (Figures 1B–1E). Four weeks later, the SADΔG-mCherry rabies virus was injected into the graft to visualize monosynaptic inputs provided by grafted DA neurons. The YFP$^+$ starter cells were seen both within the projection areas (Figures 3D, 3E–3E″) and in the transplants (Figures 3F–3F″). The starter cells were confirmed to be transplant derived based on human NCAM immunoreactivity (Figures 3D and 3E–3E‴) and of a DA phenotype based on TH expression (Figures 3F–3H).

A one in six series of coronal sections from all animals in the study was stained for mCherry using DAB immunohistochemistry (Figures 3I–3L). The mCherry$^+$ traced host cells were mapped to a rat reference atlas throughout the brain. Traced cell counts were then quantitatively compared for each functional brain region independently between the four groups of animals that received the AAV-MNM008 | DIO-Flp in either the Str or PFC (Figure 4). The counts were projected onto a 3D brain atlas (Figures 4D and 4E) and clustered based on the functional structures and nuclei (Figures 4A–4C). The counts are also displayed in the atlas as a heatmap (Figure 4F).

The input to neurons in the transplant, which had projected to A9 target regions, displayed a close similarity to

**Figure 3. AAV-based TRIO from nigral hESC-derived DA transplants**

(A) Innervation of the ipsilateral Str from DA graft transplanted to the SN (A).

(B and C) comparison between the axonal re-innervation of the PFC (A, C) between hESC grafts placed in the Str (A) and in the SN (SN, C) 26 weeks after grafting.

(D–E‴) Representative section from the PFC in SN grafted rats where afferents to A10 (VTA-type) neurons are traced. The AAV-MNM008 based DIO-Flp vector was injected into the PFC (along the dashed line in D), and the fDIO-TVA and fDIO-oG AAV vectors were injected into the graft. Both starter cells and traced cells are mCherry$^+$ (D–E′), starter cells and their projections are YFP$^+$ (D and E″), and all afferents from the transplant are hNCAM$^+$ (D and E‴).

(F–H‴) A large fraction of the starter cells was confirmed to be TH + DA neurons in the graft (F–G‴), and in the case of the PFC-injected animals, some starter cells are Calbindin$^+$ (H–H‴).

(I) The approach successfully labeled neurons with mCherry in regions expected to have monosynaptic contacts to the VTA, e.g., the PFC (I).

(J–L) In the second group of transplanted animals, we instead injected the AAV-MNM008 | DIO-Flp vector into the Str to map the afferents to the A9 type DA neurons of the transplant. In this case, we observed labeled neurons in the different regions, e.g., the motor cortex (MCx) (J), the Str (K), and the STN (L). Scale bar in C represents 50 μm in (A–C), in (D) represents 200 μm, in (E‴) represents 50 μm in (E–E‴), in (F‴) represents 200 μm in (F–F‴), in (G‴) represents 25 μm in (G–H‴), in (L) represents 200 μm in (I–L).

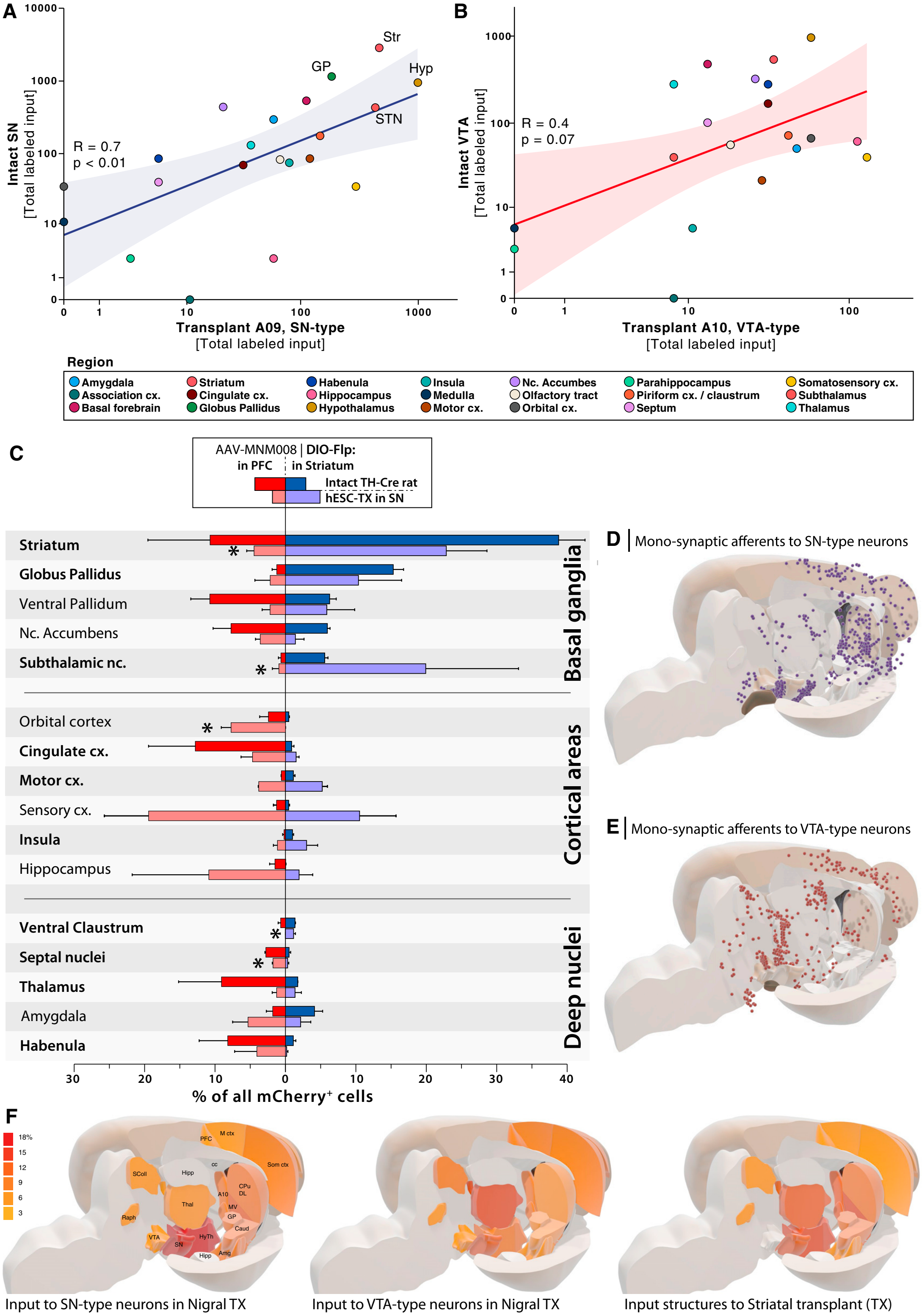


**Figure 4. Regional mapping and quantification of two-neuron tracing to nigral transplants**

(A) Correlation between host input to the SN and the A9-type neurons in the hESC-derived transplant (TX), log-log transformed plot ($R = 0.7$; $p < 0.01$).

(legend continued on next page)

the A9 population in the intact SN, as described above. All major input structures, including the Str, globus pallidus, and STN, were shared between the A9-type cells in the host SN and the graft (Figure 4A) ($R = 0.7$, $p < 0.01$). The input to the DA neurons in the graft that had projected to A10 target regions was markedly different from A9 inputs, and while it shared some input regions with the host A10 population in the host VTA, the correlation was less apparent ($R = 0.4$, $p = 0.07$) (Figure 4B). At a more granular level, the input to the A9-type neurons in the graft tracked the input to endogenous SN DA neurons in general, but with less input from the NAcc and more from the STN. Other areas from where the graft received more innervation than endogenous DA neurons include the motor and somatosensory cortical areas and the hippocampus (Figure 4C). The input to the A10-type neurons in the graft differed significantly from the A9-type neurons in the graft in several regions, including the Str, globus pallidus, STN, orbital and cingulate cortices, habenula, and septal nuclei (Figure 4C).

## DISCUSSION

In this study, we have developed a novel AAV-induced approach for two-factor neuronal connectivity that enables synaptic tracing of specific types of neurons based on their projection patterns. The tracing was achieved by combining retrogradely transported AAVs, Cre-expressing cells, and a monosynaptic recombinant rabies virus. We used the tracing approach to determine host-to-graft synaptic input of stem cell-derived Cre-expressing DA grafts and compared their inputs with those of endogenous midbrain DA neuron populations in knock-in TH-Cre rats. This approach allowed us to distinguish host input to A9-type and A10-type DA neurons within the graft based on their differential forebrain projection patterns: the A9-type neurons projected mainly to the Str, whereas the A10-type DA neurons projected to limbic and cortical areas, including the PFC. A direct comparison between these two populations of grafted DA neurons revealed that although many regions shared connectivity, several distinct regions displayed significantly different host-to-graft connectivity between the two groups. Furthermore, the connectivity profile of inputs to the graft follows, with a few exceptions, those of the corresponding regions in the intrinsic host DA system, highlighting the ability of grafted hESC-derived DA neurons to integrate within the adult mammalian brain.

The xenografting model of human DA neurons into the rat Str comes with certain limitations. The human and rodent midbrain DA circuitry have a markedly different anatomy, and differ significantly in size. Moreover, the A9 and A10 populations are not as clearly separated in the human SNpc and VTA. However, their projection patterns into the basal ganglia limbic versus motor areas seem to be preserved between species (Bjorklund and Dunnett, 2007). This is a possible explanation for why we find such striking similarities between the input to the A9-type transplanted neurons and the rat SNpc, despite the anatomical differences.

### Basal ganglia connectivity

Using the presented tracing technique, we found that the basal ganglia motor circuitry was one of the areas with the highest input to hESC-derived, midbrain-grafted DA neurons that projected to the Str. The Str, which is a central part of the basal ganglia, was one of the regions providing the most frequent synaptic inputs to grafted DA neurons that reciprocally projected there. This result closely matches both the experiments in the intact midbrain DA populations of the TH-Cre rats performed here and previous rabies-based tracing studies where both VTA and SN DA neurons receive synaptic input from the Str to a high degree (Watabe-Uchida et al., 2012). The findings indicate reciprocal communication between the grafted DA neurons and the Str. However, it remains to be shown if this reciprocal connectivity between grafted A10 and A9-like neurons and the Str is functionally similar to that between the intact Str, VTA, and SN.

The graft's overall connectivity (connections to A9- and A10-type neurons collectively) is very similar to that observed in a monosynaptic tracing study using the entire graft as potential starter cells (Adler et al., 2019; Cardoso et al., 2018; Xiong et al., 2021). However, our findings also suggest that the traced striatal connectivity input to the grafted DA neurons may display a structural specificity. This conclusion is based on the regional distribution of the mCherry$^{+}$ traced cells. A majority of labeled striatal

(B) Corresponding correlation between the input to the host VTA and A10-type neurons in the TX ($R = 0.4$; $p = 0.07$).
(C) Plotting of the number of labeled afferent neurons in each region compared between the two tracing groups and the intact dopamine system of TH-Cre$^{+}$ rats. In each brain structure (one line), the top bars (darker red and blue) represent the TH-Cre$^{+}$ intact rats, and the lower bars (lighter red and blue) represent the hESC transplanted animals. The centerline's left side contains the animals where tracing was initiated from the PFC (A10-type neurons) and the right side with tracing initiated from the Str (A9-type neurons). *A significant difference from A9-type neurons.
(D–F) Mapping of all traced neurons into a 3D brain atlas. (D) Individual monosynaptic afferent neurons to A9-type (SN) neurons. (E) Individual monosynaptic afferent neurons to A10-type (VTA) neurons. (F) The traced cells are aggregated into 3D heat maps for A9-type neurons, compared with A10-type neurons and connectivity traced from an ectopically placed transplant in the Str.

projection neurons were found in the dorsolateral Str if the tracing was initiated in A9-type grafted neurons. In contrast, projection neurons connecting to A10-type grafted neurons seemed to be more frequently in the ventral-medial Str, adjacent to the nucleus accumbens. This difference may indicate that striatal input to grafted neurons follows a specific structural organization of connectivity.

The STN was another region within the basal ganglia circuitry that was densely connected to A9-type but not to A10-type grafted neurons and to host SN neurons but not VTA neurons, indicating a clear preference for the motor circuit of the basal ganglia. The traced cells were distributed homogeneously throughout the STN without any clear anatomical division. This is also in line with previous rabies-based tracing studies in intact mice, although other studies using anterograde tracers have only identified sparse projections (Groenewegen and Berendse, 1990; Kita and Kitai, 1987; Smith et al., 1990). The STN neurons are thought to regulate nigral DA neurons through either inhibition (through excitation of inhibitory neurons in the reticulata) or facilitating burst firing through direct excitation of SN DA neurons (Smith and Grace, 1992). It is currently unknown whether grafted neurons retain this functional regulation, and this will require further study. However, both the direct synaptic inputs from the STN to the graft as well as the connectivity from the reticulata indicate that the circuitry for host-to-graft regulation remains intact.

### Cortical connectivity

The motor and somatosensory cerebral cortices were other regions that stood out in terms of monosynaptic connectivity to grafted DA neurons derived from hESCs. This connectivity differs from the host midbrain DA populations in this study. However, they are in line with previous observations using monosynaptic tracing in DAT-Cre mice restricted to the VTA or SN through stereotactic targeting (Watabe-Uchida et al., 2012) and the results obtained in previous monosynaptic tracing studies of intranigral DA neuron grafts (Adler et al., 2019; Cardoso et al., 2018; Xiong et al., 2021). The source of this discrepancy is unknown, but it may be due to the differences in the tracing methodology. The exact role of cortical motor input to A9 nigral DA neurons and their connection has not been fully explored. It is thought that cortical input is likely excitatory, given that DA neurons display increased firing in response to reward-orientated behavior (Jin and Costa, 2010).

Frontal cortices contained a small number of traced cells, which may be due to the generally small size of the grafts. Although small, there was a significant difference between input to A9-type and A10-type grafted DA neurons, with a preference for the latter. This finding is in line with that of the intact A10/VTA DA neurons. The PFC has been shown to provide significant input to VTA neurons, primarily from pyramidal layer V neurons (Gabbott et al., 2005; Sesack and Carr, 2002). The majority of traced cells in the PFC were found in either the medial PFC/anterior cingulate cortex or the orbitofrontal cortex. These two regions appear to modulate VTA neurons in a differential manner.

The refined method for tracing presented here enables selective tracing of neuronal subtypes based on their projections. Through the accurate induction of starter cells, we show a differential host-to-graft connectivity between distinct cell populations in the graft. These results suggest that much of the vital circuitry is reestablished with homotopic grafts of DA neurons. Therefore, reconstitution of the nigrostriatal pathway remains an intriguing pursuit in cell replacement therapy for Parkinson’s disease.

## EXPERIMENTAL PROCEDURES

### Monosynaptic rabies tracing

Monosynaptic rabies tracing is a powerful technique for assessing functional circuit integration (Grealish et al., 2015; Kim et al., 2016; Wickersham et al., 2007). In the context of cell replacement, the method allows a detailed assessment of the graft-to-host or host-to-graft connectivity. The approach is based on a mutated rabies virus that is rendered transmission deficient via replacement of the glycoprotein by a fluorescent marker (mCherry) and rendered transduction selective by pseudotyping the virus with an EnvA. The second part of this system is the generation of starter cells expressing the rabies glycoprotein (necessary for the rabies virus to assemble into infectious particles which can retrogradely cross the synapse). The starter cells also have to express the TVA receptor to allow the EnvA-pseudotyped rabies virus to enter the cell. The spread of the virus is limited to first-order synaptic connections. Outside a starter cell, the virus cannot assemble into infectious particles due to the lack of glycoprotein. Starter cells can readily be identified by the co-expression of mCherry (rabies) and YFP, and traced cells can be identified by expression of mCherry only.

### Research animals

All experimental procedures performed in this study were approved by the Ethical Committee to use Laboratory Animals in the Lund-Malmö region. Adult athymic nude female rats were purchased from Envigo (Hsd:RH-Foxn1$^{rnu}$), and adult female Sprague-Dawley rats (225–250 g) were purchased from Charles River (Germany). Athymic nude rats were housed in individually ventilated cages under a 12-h light/dark cycle with ad libitum access to sterile food and water. Female TH-Cre–positive Sprague-Dawley rats (Brown et al., 2013) were kept in standard cages but otherwise under identical conditions, as stated above.

### 6-OHDA lesions

Adult athymic nude female rats were lesioned using a unilateral infusion of 6-hydroxydopamine, as previously described (Heuer et al., 2013). In brief, 3μL of 6-hydroxydopamine (Sigma Aldrich) at a concentration of 3.5μg/μL (calculated from freebase weight, HBr-salt in 0.2mg/mL ascorbic acid in 0.9% sterile

saline) was injected using a 30G stainless steel cannula. The cannula was connected via polyethylene tubing to a 10-μL Hamilton syringe at a flow rate of 1 μL/min at the following coordinates (from bregma): AP, −4.0 mm; ML, −1.3 mm; and DV (from Dura), −7.0 mm with the incisor bar set at −4.5 mm. The injection needle was left in place for an additional 3 min to allow for diffusion of the toxin.

## Drug-induced rotational locomotion

The DA lesion using 6-OHDA and the maturation of the transplant were assessed using the drug-induced rotation tests (Ungerstedt et al., 1969). Results are found in Figure S4A.

Rats were placed in automated rotometer bowls modeled after the design of Ungerstedt and Arbuthnott (1970) and recorded using a rotation counting software (AccuScan Instruments Inc.). The animals were recorded for a total of 90 min after amphetamine injection (2.5 mg/kg i.p.).

## Generation of Cre-expressing hPSCs

RC17 hPSCs (Roslin Cells, hPSCreg #RCe021-A) at passage 28 were dissociated with EDTA and plated 200,000 cells/well in a 12-well plate. One hour after plating, the cells were transduced with a LV vector-expressing Cre recombinase under control of the human Synapsin-1 promoter. The cells were transduced at a multiplicity of infection of 6, which we have assessed with LV-expressed GFP to result in greater than 90% transduction. The transduced cells were expanded for nine days and then frozen in CryoStor (Sigma-Aldrich).

To ensure that the Cre-expression is stable, a vial was thawed, and the cells terminally differentiated for forty-three days *in vitro* using the protocol below. Then the cells were stained for TH and Cre.

## Cell differentiation and grafting

RC17 hPSCs were differentiated and prepared for transplantation, according to Nolbrant et al. (2017). Cultures of hPSCs were first differentiated into accurate VM progenitor patterning in N2 media with GSK3i and SHH to obtain the correct caudalization and ventralization of VM progenitors. At 9 days after the start of the differentiation protocol, the cell culture was fine tuned to the correct caudalization of early VM caudal progenitors by the addition of FGF8b. At eleven days after the start of differentiation, the cells were replated and expanded in B27 media with FGF8b, AA, and brain-derived neurotrophic factor and cultured for an additional five days *in vitro* (DIV) to give rise to late caudal VM progenitors with a high proportion positive for FOXA2/LMX1/OTX2. (See Figure S1A for details.) At 14 DIV, a subset of cells was prepared for mRNA extraction or ICC. A battery of phenotypic markers was identified using reverse transcriptase quantitative polymerase chain reaction (RT-qPCR) (Figure S1B), and the canonical marker proteins LMX1, FOXA2, and OTX2 were well-expressed (Figure S1C). The cultured cells were then prepared for transplantation by dissociation into a single-cell suspension in HBSS + DNase. Transplant surgeries of differentiated cells into the Str were performed as previously described (Grealish et al., 2015). In brief, the rats were engrafted with 150,000 hESCs at day 16 of differentiation (see below) toward a ventral midbrain DA phenotype. The cells were engrafted in a volume of 2 μL at a concentration of 75,000 cells/μL at the following coordinates (relative to bregma) into the denervated Str: AP, +0.5 mm; ML, −2.6 mm; and DV (from Dura), −4.5mm with the incisor bar set at −2.4 mm. For intranigral grafts, a volume of 2 μL at a concentration of 37,500 cells/μL was used at the following coordinates (relative to bregma): AP, −5.2 mm; ML, −2.3 mm; and DV, −7.0 mm with the incisor bar set at −4.3 mm.

## AAV production

AAV was produced as previously described (Davidsson et al., 2019; Negrini et al., 2020). In brief, HEK293T cells were seeded in 175 $cm^2$ cell culture flasks. Two hours before transfection, the medium was replaced with 27 mL fresh Dulbecco's modified Eagle medium (DMEM) supplemented with fetal bovine serum and Penicillin-Streptomycin. AAV was produced using standard polyethylenimine (PEI) transfection (Gray et al., 2011). PEI and plasmid DNA were mixed in 3 mL DMEM, incubated for 15 min, and then added to the cells. Sixteen hours after transfection, 27 mL of medium was replaced with OptiPRO serum-free medium (Thermo Fischer Scientific). AAVs were harvested 72 h after transfection using polyethylene glycol 8000 precipitation and chloroform extraction followed by phosphate-buffered saline exchange in Amicon Ultra-0.5 Centrifugal filters (Merck Millipore) (Wu et al., 2001). Purified AAVs were titered using qPCR with primers specific to the WPRE sequence. The following AAVs were produced; AAV-MNM008 | pEF1a-DIO-Flpo-WPRE-hGHpA (Addgene 87306), AAV8 | pAAV-CAG-fDIO-oG-WPRE-SV40pA (Addgene 74291), and AAV8 | CAG-FLEx(FRT)-TC (Addgene 67827) with titers ranging from 6E12 to 1.2E13 genome copies per milliliter.

## Viral surgeries

Viral injections were performed under anesthesia with a 20:1 mixture of fentanyl citrate (fentanyl) and medetomidine hypochloride (Dormitor). Coordinates for stereotactic infusions were identified relative to the bregma. A small hole was drilled through the skull, and the vector solutions were injected with a 25 μL Hamilton syringe fitted with a glass capillary (60–80 μm i.d. and 120–160 μm o.d.) and connected to an automatic infusion pump. Infusion of AAV viral vectors used 1μL of AAV-MNM008 in either the PFC AP, +3.5; ML, +0.5; DV, −4.5 or 1.5 μL; the Str AP, 0.0; ML, −3.7; or DV, −5.0 while AAVs containing constructs for Flp-induced TVA receptor, or rabies glycoprotein was injected directly into the graft: AP, −5.2; ML, −2.3; and DV, −7.0. The SADΔG-mCherry pseudotyped rabies virus was injected at two sites into the SN: (1) AP, −5.2; ML, −2.3; and DV, −7.0; and (2) AP, −4.9; ML, −2.3; and DV, −7.0 with an infusion rate of 0.2 μL/min. The capillary was left in position for two minutes before retraction, following all infusions.

## Immunohistochemistry

Animals were sacrificed seven days after rabies injection using a sodium pentobarbital overdose (Apoteksbolaget). They were transcardially perfused with 150 mL physiological saline solution followed by 250 mL of freshly prepared, ice-cold, 4% paraformaldehyde (PFA) in 0.1 M phosphate buffer (pH = 7.4). The brains were removed from the skull and postfixed for 2 h in

ice-cold PFA before storing in 25% buffered sucrose for cryoprotection, lasting at least 24 h until further processing. The brains were then cut into 35-μm coronal sections using a sliding microtome. The brain sections were collected as one in eight series and stored in antifreeze solution (0.5 M sodium phosphate buffer, 30% glycerol, and 30% ethylene glycol) at −20°C until further processing. For immunohistochemical analysis, tissue sections were washed (3×) with Tris-buffered saline (TBS) (pH 7.4) and incubated for 1 h in 3% $H_2O_2$ in 0.5% TBS Triton solution to quench endogenous peroxidase activity and to increase tissue permeability. After another washing step, the sections were blocked in 5% bovine serum, incubated for 1 h, and subsequently incubated with primary monoclonal antibodies overnight. Grafted neurons were identified through staining for anti–human-NCAM (Santa Cruz, Cat #sc-106, RRID: AB_627128, mouse monoclonal, 1:1,000), human nucleus antigen, HuNu (Sigma-Aldrich clone 235-1, Cat #MAB1281, RRID: AB_94090, mouse monoclonal, 1:200), TH (Merck Millipore, Cat #AB152, RRID: AB_390204, rabbit polyclonal, 1:1000), TH (Abcam, Cat #AB76442, RRID: AB_1524535, chicken polyclonal, 1:500), GIRK2 (Alomone labs, Cat #APC006, rabbit polyclonal, 1:80), Calbindin (Sigma-Aldrich, Cat #C9848, RRID: AB_476894, mouse monoclonal, 1:1000) FOXA2 (R&D Systems, Cat #AF2400, RRID: AB_2294104, goat polyclonal, 1:1000) while traced host neurons were identified through staining of mCherry (Scigen, Cat #AB0040-200, RRID: AB_2333092, goat polyclonal, 1:1000) and YFP (Abcam, Cat #Ab13970, RRID: AB_13970, chicken polyclonal, 1:1000). After overnight incubation, the primary antibody was first washed away using TBS (×3) and then incubated with secondary antibodies for two hours. For 3, 3′-diaminobenzidine (DAB) immunohistochemistry, biotinylated anti-mouse (BA.2001, Vector Laboratories 1:250) was used. For fluorescent immunohistochemistry, Alexa Fluor 647 Donkey anti-Mouse (Abcam, Cat #Ab150107, 1:500), Alexa Fluor 647 Donkey anti-Rabbit (Thermo Fischer Scientific, Cat #A31573, 1:500), Alexa Fluor 568 Donkey anti-Goat (Thermo Fischer Scientific, Cat #A11057, 1:500), Alexa Fluor 750 Goat anti-Rabbit (Thermo Fischer Scientific, Cat #A21039, 1:500), Alexa Fluor 488 Donkey anti-Chicken (Jackson ImmunoResearch, Cat #703-545-155, RRID: AB_2340375, 1:400) and Alexa Flour 488 Streptavidin (Jackson ImmunoResearch, Cat #016-540-084, RRID: AB_2337249, 1:400) was used. For DAB immunohistochemistry, the ABC-kit (Vectorlabs) was used after the secondary antibody incubation to amplify the staining intensity through streptavidin peroxidase conjugation followed by a DAB in 0.01% $H_2O_2$ color reaction.

For immunocytochemistry, cells were fixed in 4% PFA for 15 min in room temperature. The fixed cells were preincubated in blocking solution containing 0.1 M phosphate-buffered saline with potassium (KPBS) + 0.1% Triton +5% donkey serum for 1–3 h at room temperature. The cells were then incubated in blocking solution containing primary antibodies rabbit anti-LMX1 (1:1000, Merck Millipore, cat. no. AB10533), goat anti-FOXA2 (1:1000, R&D systems, cat. no. AF2400) and goat anti-OTX2 (1:2000, R&D Systems cat. no. AF1979) overnight at 4°C and the following day washed three times with KBPS. Secondary fluorophore-conjugated antibodies (1:200, Jackson ImmunoResearch Laboratories) and DAPI (1:500) was added to the cells in the blocking solution for 2 h in room temperature and the cells where then finally washed three times with KPBS.

### qRT PCR

RNA was isolated using the RNeasy Microkit (Qiagen) and reverse transcription was performed using the Maxima First Strand cDNA Synthesis Kit for RT-qPCR (Thermo Fisher Scientific), with 1 μg of extracted RNA. The cDNA (1 μL) was then mixed with the relevant primers (4 μL; Integrated DNA Technologies) and LightCycler 480 SYBR Green I Master (5 μL, Roche) using a Bravo pipetting robot instrument (Agilent) and analyzed by quantitative RT-PCR on a LightCycler 480 II instrument (Roche) using a 40× cycle two-step protocol with a 60°C, 1-min annealing/elongation step and a 95°C, and a 30-s denaturation step. The average cycle threshold (CT) values were calculated from three technical replicates and were used to determine the relative gene expression using the ΔΔCT method. The average fold change was based on two different housekeeping genes (ACTB and GAPDH) and the relative gene expression is described relative to undifferentiated hESCs.

### Analysis of traced neurons

Immunolabled mCherry brain sections mounted on glass slides were scanned using a rebuilt flatbed scanner (Epson Perfection v750 Pro) at 6400 dpi resolution. Based on The Rat Brain Atlas, seventh edition, each digitally scanned section was placed into the corresponding coronal section, ensuring accurate localization of each traced neuron. The tracing of neurons was then conducted by meticulous light microscopy analysis, and each mCherry$^+$ neuron was marked into the correct location according to the scanned section/atlas overlay.

The 3D template of the rat brain used in this study for mapping of ΔG-rabies traced neurons was built using 3D software (Cinema 4D), with structural proportions based on the Paxinos and Watson rat brain atlas (Paxinos and Watson, 2005). To represent host synaptic inputs to the graft, the mapped neurons from mCherry DAB-stained sections were transferred into the 3D space, with each dot representing the location of an mCherry$^+$ neuron.

Statistics were performed using R statistics package version 3.2 and IBM SPSS version 27. Correlation coefficients in Figures 4A and 4B were calculated using Spearman's rho nonparametric test. Differences in the number of traced neurons in each region were assessed using the Mann–Whitney *U* nonparametric test.

## SUPPLEMENTAL INFORMATION

Supplemental information can be found online at https://doi.org/10.1016/j.stemcr.2021.11.014.

## AUTHOR CONTRIBUTIONS

Conceptualization TB, MP. Formal Analysis PAK, TB. Investigation PAK, TC, MÅ, SN, AFA, XL, Methodology TB, MP, MD, AH.

Visualization TB, PAK, MÅ. Writing – original draft TB, PAK. Writing – review and editing TB, PAK, MP, MÅ, AH, AFA, MD.

## CONFLICTS OF INTEREST

MD and TB are inventors of multiple patents related to gene therapy. MD and TB are founders and directors of Brave Bioscience AB. TB is a co-founder and SAB member of Dyno Therapeutics. The remaining authors declare no competing interests.

## ACKNOWLEDGMENTS

The authors would like to thank Bengt Mattsson for expert assistance in 3D atlas generation and 3D mapping of *traced cells* and Agnete Kirkeby for the generation of the Cre-expressing hESCs. The research leading to these results has received funding from the New York Stem Cell Foundation, European Research Council (ERC) under ERC Grant Agreement 771427, Swedish Research Council (2016-00873, 2016-01997, and 2020-01684); Knut & Alice Wallenberg Foundation (KAW 2018-0040); Swedish Parkinson Foundation; Swedish Brain Foundation, Crafoord foundation; Strategic Research Area at Lund University Multipark and the Segerfalk foundation. MP is a New York Stem Cell Foundation Robertson Investigator. This study was supported by the European Union–funded project NSC-Reconstruct (European Union, H2020, GA no 874758, 2020-23). pEF1a-DIO-Flp-WPRE-hGHpA was a gift from Li Zhang (Addgene plasmid # 87306) pAAV-CAG-fDIO-oG-WPRE-SV40pA was a gift from Edward Callaway (Addgene plasmid # 74291), and CAG-FLEx(FRT)-TC was a gift from Liqun Luo (Addgene plasmid # 67827).

Received: June 20, 2021
Revised: November 25, 2021
Accepted: November 26, 2021
Published: December 30, 2021

## REFERENCES

Adler, A.F., Cardoso, T., Nolbrant, S., Mattsson, B., Hoban, D.B., Jarl, U., Wahlestedt, J.N., Grealish, S., Bjorklund, A., and Parmar, M. (2019). hESC-derived dopaminergic transplants integrate into basal Ganglia circuitry in a preclinical model of Parkinson's disease. Cell. Rep. *28*, 3462–3473.e3465. https://doi.org/10.1016/j.celrep.2019.08.058.

Aldrin-Kirk, P., Heuer, A., Wang, G., Mattsson, B., Lundblad, M., Parmar, M., and Bjorklund, T. (2016). DREADD modulation of transplanted DA neurons reveals a novel parkinsonian dyskinesia mechanism mediated by the serotonin 5-HT6 receptor. Neuron *90*, 955–968. https://doi.org/10.1016/j.neuron.2016.04.017.

Barker, R.A., Drouin-Ouellet, J., and Parmar, M. (2015). Cell-based therapies for Parkinson disease-past insights and future potential. Nat. Rev. Neurol. *11*, 492–503. https://doi.org/10.1038/nrneurol.2015.123.

Bjorklund, A., and Dunnett, S.B. (2007). Dopamine neuron systems in the brain: an update. Trends Neurosci. *30*, 194–202. https://doi.org/10.1016/j.tins.2007.03.006.

Bjorklund, A., and Parmar, M. (2020). Neuronal replacement as a tool for basal ganglia circuitry repair: 40 years in perspective. Front. Cell. Neurosci. *14*, 146. https://doi.org/10.3389/fncel.2020.00146.

Brown, A.J., Fisher, D.A., Kouranova, E., McCoy, A., Forbes, K., Wu, Y., Henry, R., Ji, D., Chambers, A., Warren, J., et al. (2013). Whole-rat conditional gene knockout via genome editing. Nat. Methods *10*, 638–640. https://doi.org/10.1038/nmeth.2516.

Cardoso, T., Adler, A.F., Mattsson, B., Hoban, D.B., Nolbrant, S., Wahlestedt, J.N., Kirkeby, A., Grealish, S., Bjorklund, A., and Parmar, M. (2018). Target-specific forebrain projections and appropriate synaptic inputs of hESC-derived dopamine neurons grafted to the midbrain of parkinsonian rats. J. Comp. Neurol. *526*, 2133–2146. https://doi.org/10.1002/cne.24500.

Chen, Y., Xiong, M., Dong, Y., Haberman, A., Cao, J., Liu, H., Zhou, W., and Zhang, S.C. (2016). Chemical control of grafted human PSC-derived neurons in a mouse model of Parkinson's disease. Cell Stem Cell *18*, 817–826. https://doi.org/10.1016/j.stem.2016.03.014.

Davidsson, M., Wang, G., Aldrin-Kirk, P., Cardoso, T., Nolbrant, S., Hartnor, M., Mudannayake, J., Parmar, M., and Bjorklund, T. (2019). A systematic capsid evolution approach performed in vivo for the design of AAV vectors with tailored properties and tropism. Proc. Natl. Acad. Sci. U S A https://doi.org/10.1073/pnas.1910061116.

Doi, D., Magotani, H., Kikuchi, T., Ikeda, M., Hiramatsu, S., Yoshida, K., Amano, N., Nomura, M., Umekage, M., Morizane, A., and Takahashi, J. (2020). Pre-clinical study of induced pluripotent stem cell-derived dopaminergic progenitor cells for Parkinson's disease. Nat. Commun. *11*, 3369. https://doi.org/10.1038/s41467-020-17165-w.

Gabbott, P.L., Warner, T.A., Jays, P.R., Salway, P., and Busby, S.J. (2005). Prefrontal cortex in the rat: projections to subcortical autonomic, motor, and limbic centers. J. Comp. Neurol. *492*, 145–177. https://doi.org/10.1002/cne.20738.

Gray, S.J., Choi, V.W., Asokan, A., Haberman, R.A., McCown, T.J., and Samulski, R.J. (2011). Production of recombinant adeno-associated viral vectors and use in in vitro and in vivo administration. Curr. Protoc. Neurosci. Chapter 4, Unit 4 17. https://doi.org/10.1002/0471142301.ns0417s57.

Grealish, S., Diguet, E., Kirkeby, A., Mattsson, B., Heuer, A., Bramoulle, Y., Van Camp, N., Perrier, A.L., Hantraye, P., Bjorklund, A., and Parmar, M. (2014). Human ESC-derived dopamine neurons show similar preclinical efficacy and potency to fetal neurons when grafted in a rat model of Parkinson's disease. Cell Stem Cell *15*, 653–665. https://doi.org/10.1016/j.stem.2014.09.017.

Grealish, S., Heuer, A., Cardoso, T., Kirkeby, A., Jonsson, M., Johansson, J., Bjorklund, A., Jakobsson, J., and Parmar, M. (2015). Monosynaptic tracing using modified rabies virus reveals early and extensive circuit integration of human embryonic stem cell-derived neurons. Stem Cell Reports *4*, 975–983. https://doi.org/10.1016/j.stemcr.2015.04.011.

Grealish, S., Jonsson, M.E., Li, M., Kirik, D., Bjorklund, A., and Thompson, L.H. (2010). The A9 dopamine neuron component in grafts of ventral mesencephalon is an important determinant

for recovery of motor function in a rat model of Parkinson's disease. Brain *133*, 482–495. https://doi.org/10.1093/brain/awp328.

Groenewegen, H.J., and Berendse, H.W. (1990). Connections of the subthalamic nucleus with ventral striatopallidal parts of the basal ganglia in the rat. J. Comp. Neurol. *294*, 607–622. https://doi.org/10.1002/cne.902940408.

Heuer, A., Lelos, M.J., Kelly, C.M., Torres, E.M., and Dunnett, S.B. (2013). Dopamine-rich grafts alleviate deficits in contralateral response space induced by extensive dopamine depletion in rats. Exp. Neurol. *247*, 485–495. https://doi.org/10.1016/j.expneurol.2013.01.020.

Jin, X., and Costa, R.M. (2010). Start/stop signals emerge in nigrostriatal circuits during sequence learning. Nature *466*, 457–462. https://doi.org/10.1038/nature09263.

Kim, E.J., Jacobs, M.W., Ito-Cole, T., and Callaway, E.M. (2016). Improved monosynaptic neural circuit tracing using engineered rabies virus glycoproteins. Cell Rep. *15*, 692–699. https://doi.org/10.1016/j.celrep.2016.03.067.

Kim, T.W., Koo, S.Y., and Studer, L. (2020). Pluripotent stem cell therapies for Parkinson disease: present challenges and future opportunities. Front. Cell Dev. Biol. *8*, 729. https://doi.org/10.3389/fcell.2020.00729.

Kim, T.W., Piao, J., Koo, S.Y., Kriks, S., Chung, S.Y., Betel, D., Socci, N.D., Choi, S.J., Zabierowski, S., Dubose, B.N., et al. (2021). Biphasic activation of WNT signaling facilitates the derivation of midbrain dopamine neurons from hESCs for translational use. Cell Stem Cell *28*, 343–355.e345. https://doi.org/10.1016/j.stem.2021.01.005.

Kirkeby, A., Grealish, S., Wolf, D.A., Nelander, J., Wood, J., Lundblad, M., Lindvall, O., and Parmar, M. (2012). Generation of regionally specified neural progenitors and functional neurons from human embryonic stem cells under defined conditions. Cell Rep. *1*, 703–714. https://doi.org/10.1016/j.celrep.2012.04.009.

Kirkeby, A., Nolbrant, S., Tiklova, K., Heuer, A., Kee, N., Cardoso, T., Ottosson, D.R., Lelos, M.J., Rifes, P., Dunnett, S.B., et al. (2017). Predictive markers guide differentiation to improve graft outcome in clinical translation of hESC-based therapy for Parkinson's disease. Cell Stem Cell *20*, 135–148. https://doi.org/10.1016/j.stem.2016.09.004.

Kita, H., and Kitai, S.T. (1987). Efferent projections of the subthalamic nucleus in the rat: light and electron microscopic analysis with the PHA-L method. J. Comp. Neurol. *260*, 435–452. https://doi.org/10.1002/cne.902600309.

Kriks, S., Shim, J.W., Piao, J., Ganat, Y.M., Wakeman, D.R., Xie, Z., Carrillo-Reid, L., Auyeung, G., Antonacci, C., Buch, A., et al. (2011). Dopamine neurons derived from human ES cells efficiently engraft in animal models of Parkinson's disease. Nature *480*, 547–551. https://doi.org/10.1038/nature10648.

Negrini, M., Wang, G., Heuer, A., Bjorklund, T., and Davidsson, M. (2020). AAV production everywhere: a simple, fast, and reliable protocol for in-house AAV vector production based on chloroform extraction. Curr. Protoc. Neurosci. *93*, e103. https://doi.org/10.1002/cpns.103.

Niclis, J.C., Gantner, C.W., Alsanie, W.F., McDougall, S.J., Bye, C.R., Elefanty, A.G., Stanley, E.G., Haynes, J.M., Pouton, C.W., Thompson, L.H., and Parish, C.L. (2017). Efficiently specified ventral midbrain dopamine neurons from human pluripotent stem cells under xeno-free conditions restore motor deficits in Parkinsonian rodents. Stem Cells Transl. Med. *6*, 937–948. https://doi.org/10.5966/sctm.2016-0073.

Nolbrant, S., Heuer, A., Parmar, M., and Kirkeby, A. (2017). Generation of high-purity human ventral midbrain dopaminergic progenitors for in vitro maturation and intracerebral transplantation. Nat. Protoc. *12*, 1962–1979. https://doi.org/10.1038/nprot.2017.078.

Parmar, M., Grealish, S., and Henchcliffe, C. (2020). The future of stem cell therapies for Parkinson disease. Nat. Rev. Neurosci. *21*, 103–115. https://doi.org/10.1038/s41583-019-0257-7.

Paxinos, G., and Watson, C. (2005). The Rat Brain in Stereotaxic Coordinates, 5th ed. (Academic Press).

Reyes, S., Fu, Y., Double, K., Thompson, L., Kirik, D., Paxinos, G., and Halliday, G.M. (2012). GIRK2 expression in dopamine neurons of the substantia nigra and ventral tegmental area. J. Comp. Neurol. *520*, 2591–2607. https://doi.org/10.1002/cne.23051.

Schwarz, L.A., Miyamichi, K., Gao, X.J., Beier, K.T., Weissbourd, B., DeLoach, K.E., Ren, J., Ibanes, S., Malenka, R.C., Kremer, E.J., and Luo, L. (2015). Viral-genetic tracing of the input-output organization of a central noradrenaline circuit. Nature *524*, 88–92. https://doi.org/10.1038/nature14600.

Sesack, S.R., and Carr, D.B. (2002). Selective prefrontal cortex inputs to dopamine cells: implications for schizophrenia. Physiol. Behav. *77*, 513–517.

Smith, I.D., and Grace, A.A. (1992). Role of the subthalamic nucleus in the regulation of nigral dopamine neuron activity. Synapse *12*, 287–303. https://doi.org/10.1002/syn.890120406.

Smith, Y., Hazrati, L.N., and Parent, A. (1990). Efferent projections of the subthalamic nucleus in the squirrel monkey as studied by the PHA-L anterograde tracing method. J. Comp. Neurol. *294*, 306–323. https://doi.org/10.1002/cne.902940213.

Steinbeck, J.A., Choi, S.J., Mrejeru, A., Ganat, Y., Deisseroth, K., Sulzer, D., Mosharov, E.V., and Studer, L. (2015). Optogenetics enables functional analysis of human embryonic stem cell-derived grafts in a Parkinson's disease model. Nat. Biotechnol. *33*, 204–209. https://doi.org/10.1038/nbt.3124.

Takahashi, J. (2020). iPS cell-based therapy for Parkinson's disease: a Kyoto trial. Regen. Ther. *13*, 18–22. https://doi.org/10.1016/j.reth.2020.06.002.

Ungerstedt, U., and Arbuthnott, G.W. (1970). Quantitative recording of rotational behavior in rats after 6-hydroxy-dopamine lesions of the nigrostriatal dopamine system. Brain Res. *24*, 485–493.

Ungerstedt, U., Butcher, L.L., Butcher, S.G., Anden, N.E., and Fuxe, K. (1969). Direct chemical stimulation of dopaminergic mechanisms in the neostriatum of the rat. Brain Res. *14*, 461–471.

Watabe-Uchida, M., Zhu, L., Ogawa, S.K., Vamanrao, A., and Uchida, N. (2012). Whole-brain mapping of direct inputs to midbrain dopamine neurons. Neuron *74*, 858–873. https://doi.org/10.1016/j.neuron.2012.03.017.

Wickersham, I.R., Finke, S., Conzelmann, K.K., and Callaway, E.M. (2007). Retrograde neuronal tracing with a deletion-mutant rabies virus. Nat. Methods *4*, 47–49. https://doi.org/10.1038/nmeth999.

Wu, X., Dong, X., Wu, Z., Cao, H., Niu, D., Qu, J., Wang, H., and Hou, Y. (2001). A novel method for purification of recombinant adenoassociated virus vectors on a large scale. Chin. Sci. Bull. *46*, 485–488.

Xiong, M., Tao, Y., Gao, Q., Feng, B., Yan, W., Zhou, Y., Kotsonis, T.A., Yuan, T., You, Z., Wu, Z., et al. (2021). Human stem cell-derived neurons repair circuits and restore neural function. Cell Stem Cell *28*, 112–126.e6. https://doi.org/10.1016/j.stem.2020.08.014.

**Stem Cell Reports, Volume *17***

## Supplemental Information

# A novel two-factor monosynaptic TRIO tracing method for assessment of circuit integration of hESC-derived dopamine transplants

**Patrick Aldrin-Kirk, Malin Åkerblom, Tiago Cardoso, Sara Nolbrant, Andrew F. Adler, Xiaohe Liu, Andreas Heuer, Marcus Davidsson, Malin Parmar, and Tomas Björklund**

# **Supplement** | A novel two-factor monosynaptic TRIO tracing method for assessment of circuit integration of hESC-derived dopamine transplants


## Author list

Patrick Aldrin-Kirk[1,3], Malin Åkerblom[1,3], Tiago Cardoso[2,3], Sara Nolbrant[2,3], Andrew F. Adler[2,3], Xiaohe Liu[1,3], Andreas Heuer[1,3], Marcus Davidsson[1,3], Malin Parmar[2,3], and Tomas Björklund[1,3*]

## Affiliations

1. Molecular Neuromodulation, Department of Experimental Medical Science, Lund University, 221 84 Lund, Sweden

2. Developmental and Regenerative Neurobiology, Department of Experimental Medical Science, Lund University, 221 84 Lund, Sweden

3. Wallenberg Neuroscience Center, Lund University, Lund Sweden

## Corresponding Author

* Tomas Björklund, Molecular Neuromodulation, Lund University, BMC A10 22184, Lund Sweden. E-mail: tomas.bjorklund@med.lu.se

## Supplemental figures

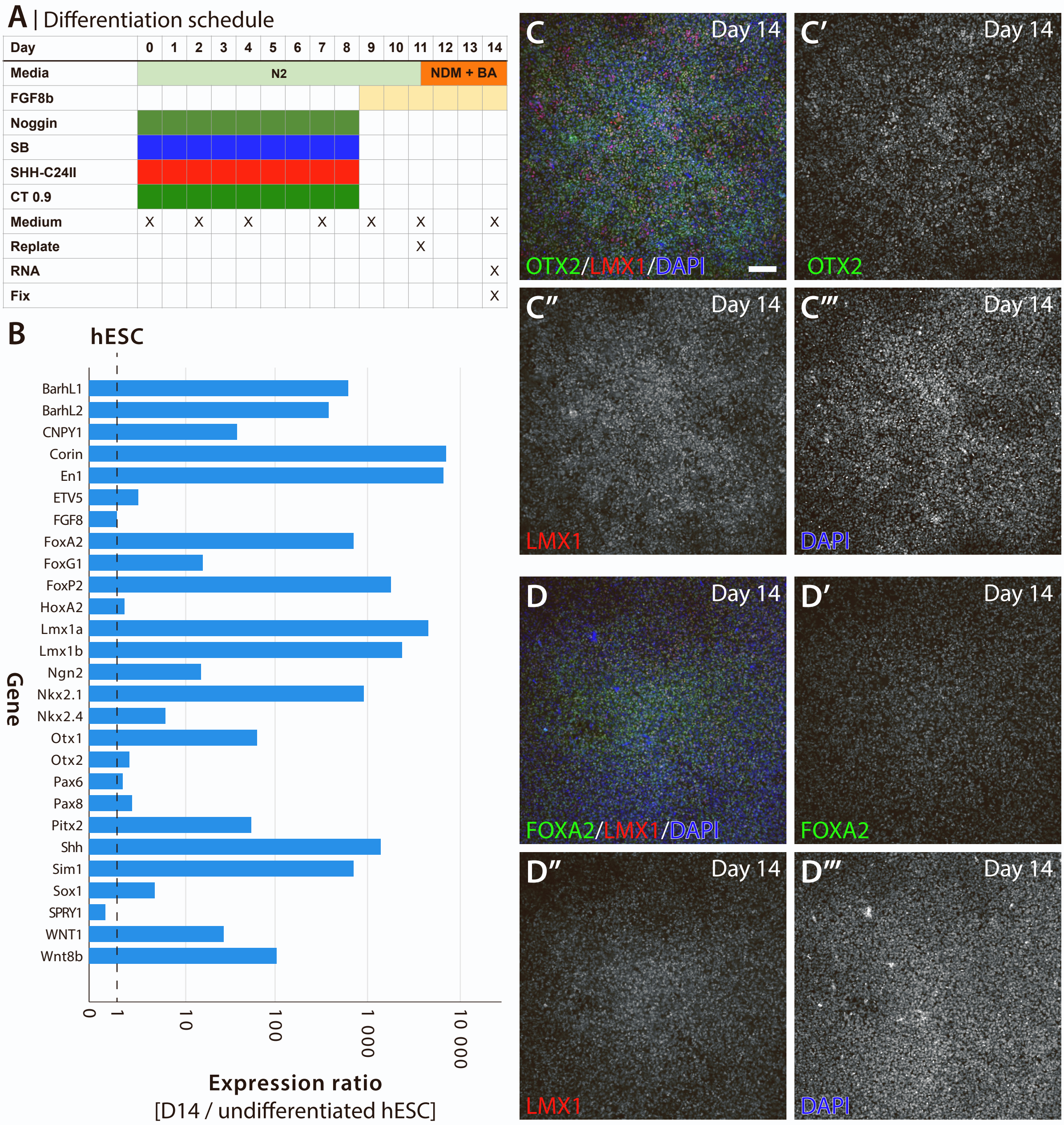


**Figure S1** | Validation of the hESC differentiation protocol on LV-Cre transduced RC17 cell line, related to Figure 1

(**A**) Differentiation schedule, (**B**), quantitative RT-PCR of phenotypic markers conducted day 14 of the differentiation protocol. Transcript expression is normalized against ACTB and GAPDH and presented as fold change over undifferentiated RC17 hESCs. (**C-D**), Immunohistochemical detection of the phenotypic markers LMX1, OTX2, FOXA2 on cells fixed by Day 14 of the differentiation protocol. Scale bar in C represents 100 µm in C-D'''.

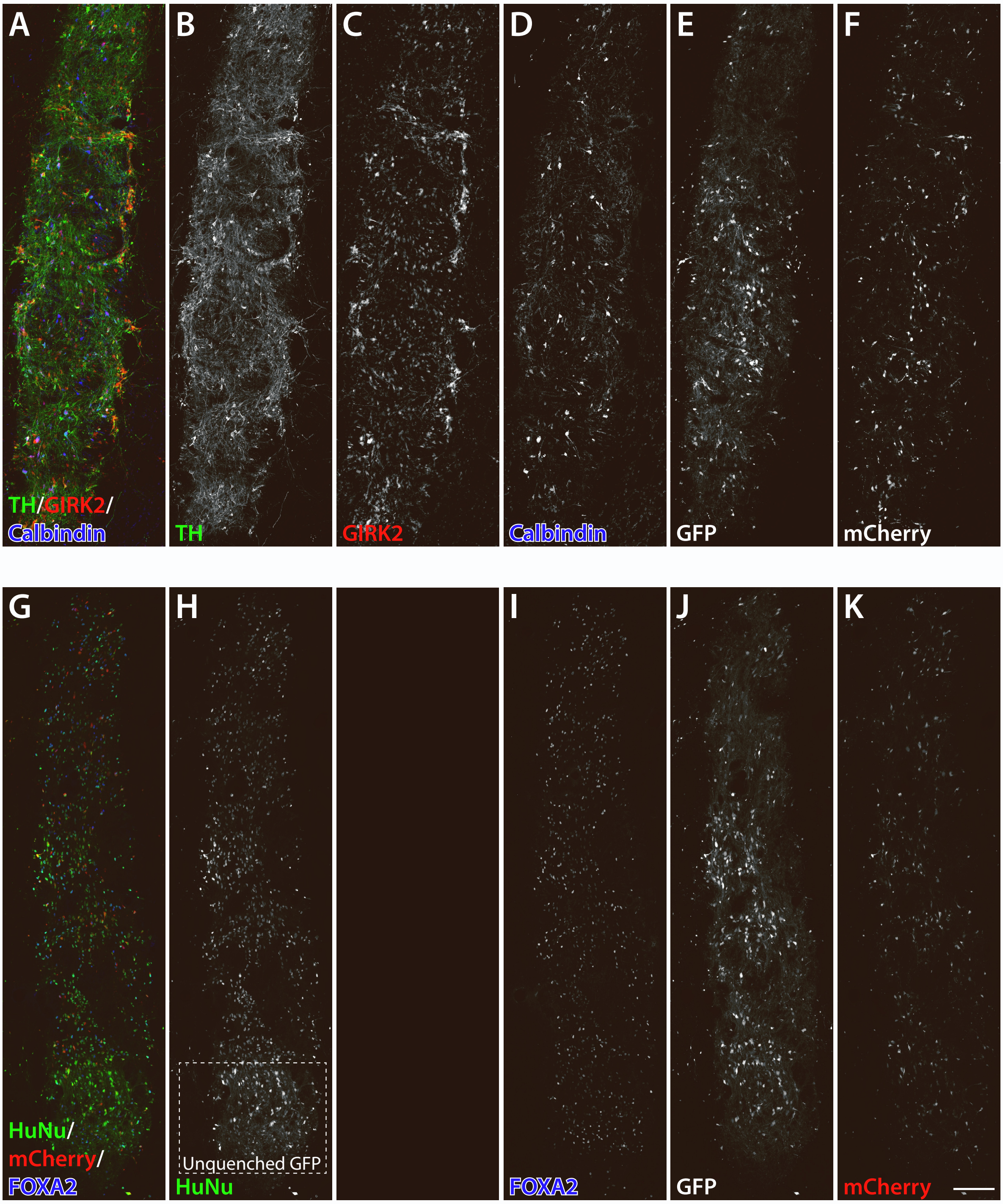


**Figure S2 |** Validation of phenotypic markers on matured striatal transplants, related to Figure 1

Immunohistochemical characterization phenotype and maturation of an hESC-derived graft placed in the striatum. To allow for additional fluorescent markers, biotinylated secondary Abs were used against TH (B) and HuNu (H). After the first round of imaging, the endogenous GFP (E, J) was quenched using high power 488nm LED light. After that, the sections were incubated with streptavidin-Alexa488 and re-imaged. A small region was retained unquenched for reference (dashed line in H). Scale bar in K represents 200µm.

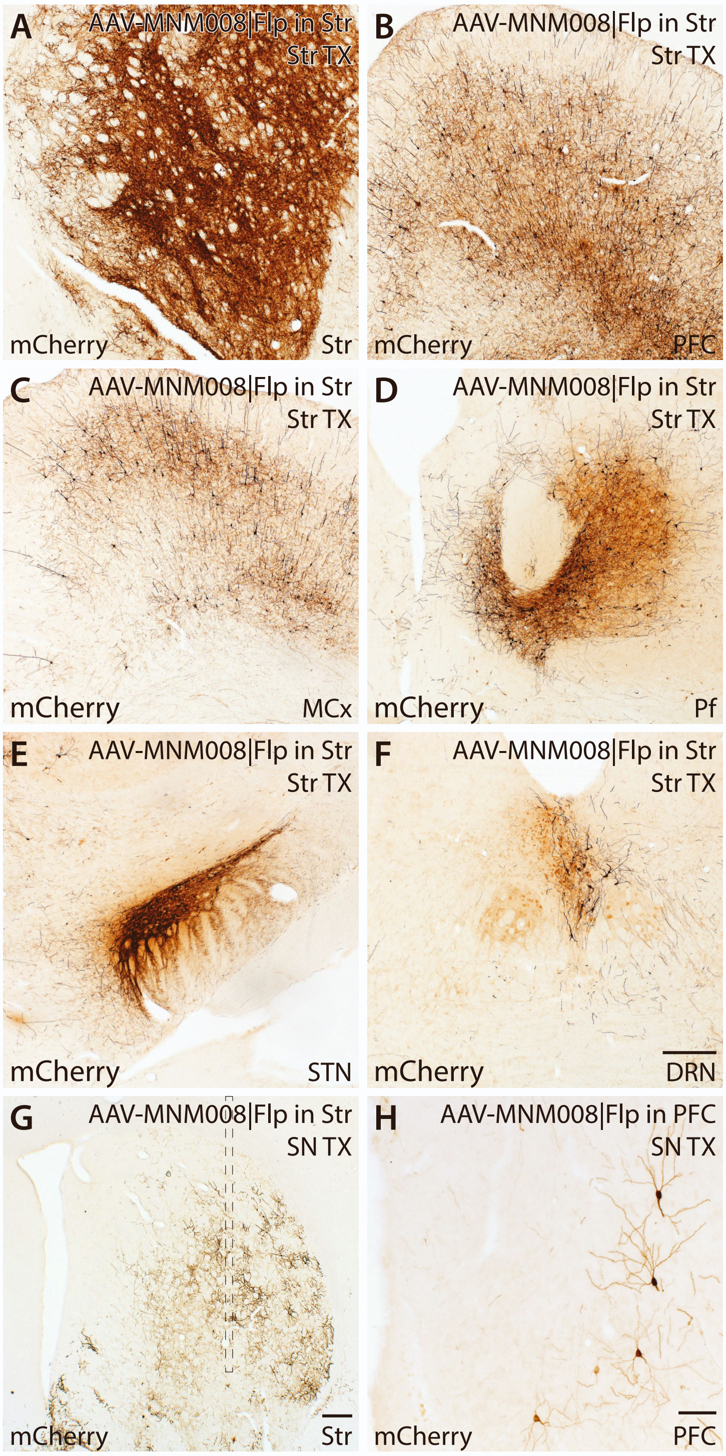


**Figure S3** | Validation of AAV-based TRIO hESC-derived DA transplants in the striatum, related to Figure 1

Input to the striatal DA transplants is found in the ipsilateral striatum (Str, **A**), prefrontal cortex (PFC, **B**), the motor cortex (MCx, **C**), parafascicular nucleus (Pf, **D**), subthalamic nucleus (STN, **E**), and the dorsal Raphe nucleus (DRN, **F**). (**G**) Validation of the AAV-induced TRIO in the endogenous DA system. Injection shown as a dashed line. (**H**) Traced input nigral DA transplant with DIO-Flp injected into the PFC. Scale bar in F represents 200µm in A-F, in G represents 500µm, in H represents 50 µm.

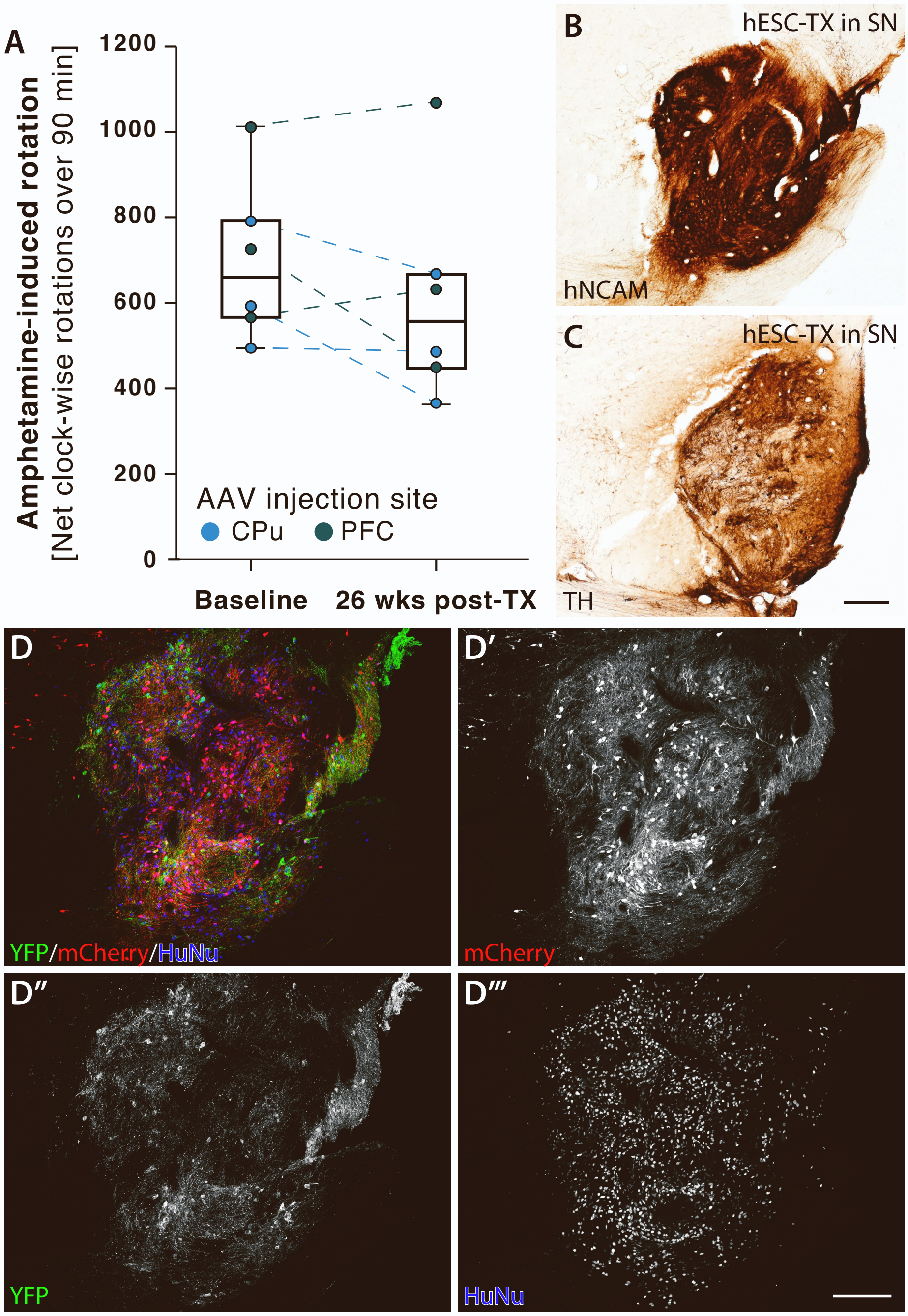


**Figure S4 |** Histology of nigral hESC-derived DA transplants and AAV-induced TRIO, related to Figure 3

(A) Amphetamine-induced rotation at Baseline (pre-transplantation) and 26wks post-transplantation. (**B**) Human NCAM labels all surviving hESC-derived cells in the transplant (TX). (C) A significant fraction of the surviving neurons are dopaminergic, identified using IHC against tyrosine hydroxylase (TH). (D-D'''). All YFP+ starter neurons are confirmed to be of human origin through the co-labeling with the human nuclei (HuNu) antibody. Scale bar in C represents 200µm in B-C, in D''' represents 200µm, in D-D'''.

## Supplemental table 1 | qPCR primers, related to Figure 1

| **Gene** | **Full gene name** | **Primer sequence (fwd/rev)** |
|---|---|---|
| *ACTB* | beta-actin | CCTTGCACATGCCGGAG |
| | | GCACAGAGCCTCGCCTT |
| *BARHL1* | BarH like homeobox 1 | GTACCAGAACCGCAGGACTAAA |
| | | AGAAATAAGGCGACGGGAACAT |
| *BARHL2* | BarH like homeobox 2 | GGAGATTACGAGTAGCCGTGAG |
| | | AAGCTACGCTCCAGTTGATTGA |
| *CNPY1* | Canopy FGF signaling regulator 1 | TTGGCCTCTCAAACACCATTCT |
| | | GAGCGAAACAAAACGCAATCAC |
| *CORIN* | Corin, serine peptidase | CATATCTCCATCGCCTCAGTTG |
| | | GGCAGGAGTCCATGACTGT |
| *EN1* | Engrailed 1 | CGTGGCTTACTCCCCATTTA |
| | | TCTCGCTGTCTCTCCCTCTC |
| *ETV5* | ETS variant 5 | TCATCCTACATGAGAGGGGGTT |
| | | GACTTTGCCTTCCAGTCTCTCA |
| *FGF8* | Fibroblast growth factor 8 | ACAGCGCTGCAGAATGCCAAGT |
| | | GAAGTGGACCTCACGCTGGTGC |
| *FOXA2* | Forkhead box A2 | CCGTTCTCCATCAACAACCT |
| | | GGGGTAGTGCATCACCTGTT |
| *FOXG1* | Forkhead box G1 | TGGCCCATGTCGCCCTTCCT |
| | | GCCGACGTGGTGCCGTTGTA |
| *FOXP2* | Forkhead box P2 | ATGAGCACTCTAAGCAGCCAAT |
| | | GTTGCAGATGCAGCAGTTCTAC |
| *GAPDH* | Glyceraldehyde-3-phosphate dehydrogenase | TTGAGGTCAATGAAGGGGTC |
| | | GAAGGTGAAGGTCGGAGTCA |
| *HOXA2* | Homeobox A2 | CGTCGCTCGCTGAGTGCCTG |
| | | TGTCGAGTGTGAAAGCGTCGAGG |
| *LMX1A* | LIM homeobox transcription factor a | CGCATCGTTTCTTCTCCTCT |
| | | CAGACAGACTTGGGGCTCAC |
| *LMX1B* | LIM homeobox transcription factor b | CTTAACCAGCCTCAGCGACT |
| | | TCAGGAGGCGAAGTAGGAAC |
| *NEUROG2* | Neurogenin 2 | ATCCGAGCAGCACTAACACG |
| | | GCACAGGCCAAAGTCACAG |
| *NKX2-1* | NK2 homeobox 1 | AGGGCGGGGCACAGATTGGA |
| | | GCTGGCAGAGTGTGCCCAGA |
| *NKX2-4* | NK2 homeobox 4 | AACTGCGATTCAAAACGAACCG |
| | | GCCTCGTGGCATAATGTTACAC |
| *OTX2* | Orthodenticle homeobox 2 | ACAAGTGGCCAATTCACTCC |
| | | GAGGTGGACAAGGGATCTGA |
| *OTX1* | Orthodenticle homeobox 1 | TATAAGGACCAAGCCTCATGGC |
| | | TTCTCCTCTTTCATTCCTGGGC |
| *PAX6* | Paired box 6 | TGGTATTCTCTCCCCCTCCT |
| | | TAAGGATGTTGAACGGGCAG |
| *PAX8* | Paired box 8 | ATAGCTGCCGACTAAGCATTGA |
| | | ATCCGTGCGAAGGTGCTTT |
| *PITX2* | Paired-like homeodomain 2 | AACTCTATGAACGTCAACCCCC |
| | | CGACATGCTCATGGACGAGATA |
| *SHH* | Sonic hedgehog | CCAATTACAACCCCGACATC |
| | | AGTTTCACTCCTGGCCACTG |
| *SIM1* | Single-minded family bHLH transcription factor 1 | AAAGGGGGCCAAATCCCGGC |
| | | TCCGCCCCACTGGCTGTCAT |
| *SOX1* | SRY-box 1 | GGGAAAACGGGCAAAATAAT |
| | | TTTTGCGTTCACATCGGTTA |
| *SPRY1* | Sprouty RTK signaling antagonist 1 | GCCCTGGATAAGGAACAGCTAC |
| | | GCCGAAATGCCTAATGCAAAGA |
| *WNT1* | Wnt family member 1 | GAGCCACGAGTTTGGATGTT |
| | | TGCAGGGAGAAAGGAGAGAA |
| *WNT8B* | Wnt family member 8B | CTAGTGGGGCAATGACTTTCCT |
| | | TTCCTAGACCTTCGGGGTATGT |
